# Wastewater surveillance provides 10-days forecasting of COVID-19 hospitalizations superior to cases and test positivity: A prediction study

**DOI:** 10.1016/j.idm.2023.10.004

**Published:** 2023-10-31

**Authors:** Dustin T. Hill, Mohammed A. Alazawi, E. Joe Moran, Lydia J. Bennett, Ian Bradley, Mary B. Collins, Christopher J. Gobler, Hyatt Green, Tabassum Z. Insaf, Brittany Kmush, Dana Neigel, Shailla Raymond, Mian Wang, Yinyin Ye, David A. Larsen

**Affiliations:** a Department of Public Health, Syracuse University, Syracuse, NY, 13244, USA; b Center for Environmental Health, New York State Department of Health, Albany, NY, USA; c CDC Foundation, Atlanta, GA, USA; d Department of Civil, Structural and Environmental Engineering, University at Buffalo, Buffalo, NY, USA; e School of Marine and Atmospheric Sciences, Sustainability Studies Division, Stony Brook University, Stony Brook, NY, USA; f Institute for Advanced Computational Science, Stony Brook University, Stony Brook, NY, USA; g New York State Center for Clean Water Technology, Stony Brook University, Stony Brook, NY, USA; h School of Marine and Atmospheric Sciences, Stony Brook University, Stony Brook, NY, USA; i Department of Environmental Biology, State University of New York College of Environmental Science and Forestry, Syracuse, NY, USA; j Department of Epidemiology and Biostatistics, School of Public Health, University at Albany, Rensselaer, NY, USA; k Department of Civil Engineering, College of Engineering and Applied Sciences, Stony Brook University, Stony Brook, NY, USA

**Keywords:** COVID-19 hospitalizations, Wastewater-based epidemiology, Forecasting, Prediction, SARS-CoV-2

## Abstract

**Background:**

The public health response to COVID-19 has shifted to reducing deaths and hospitalizations to prevent overwhelming health systems. The amount of SARS-CoV-2 RNA fragments in wastewater are known to correlate with clinical data including cases and hospital admissions for COVID-19. We developed and tested a predictive model for incident COVID-19 hospital admissions in New York State using wastewater data.

**Methods:**

Using county-level COVID-19 hospital admissions and wastewater surveillance covering 13.8 million people across 56 counties, we fit a generalized linear mixed model predicting new hospital admissions from wastewater concentrations of SARS-CoV-2 RNA from April 29, 2020 to June 30, 2022. We included covariates such as COVID-19 vaccine coverage in the county, comorbidities, demographic variables, and holiday gatherings.

**Findings:**

Wastewater concentrations of SARS-CoV-2 RNA correlated with new hospital admissions per 100,000 up to ten days prior to admission. Models that included wastewater had higher predictive power than models that included clinical cases only, increasing the accuracy of the model by 15%. Predicted hospital admissions correlated highly with observed admissions (r = 0.77) with an average difference of 0.013 hospitalizations per 100,000 (95% CI = [0.002, 0.025])

**Interpretation:**

Using wastewater to predict future hospital admissions from COVID-19 is accurate and effective with superior results to using case data alone. The lead time of ten days could alert the public to take precautions and improve resource allocation for seasonal surges.

## 1 Introduction

The COVID-19 pandemic overwhelmed global hospital bed capacity, (Miller et al., 2020) worsening case outcomes and causing greater case-fatality among both COVID-19 hospitalizations and individuals hospitalized for other conditions (Khera et al., 2021). Vaccination efforts have markedly reduced adverse outcomes including hospitalization, however vaccine coverage remains sub-optimal (Moghadas et al., 2021) and the health system has suffered numerous surges of COVID-19 hospitalizations following the rollout of COVID-19 vaccines. People living with comorbidities remain at elevated risk, and restricted resources, staffing shortages and reduced capacity of hospital systems could still contribute to adverse outcomes (Sono-Setati et al., 2022). Considering this, it is imperative that methods of hospitalization forecasting are improved to predict disease burden accurately during anticipated surges of COVID-19 transmission. Current hospitalization forecasting methodology relies heavily on clinical data, (Morozova et al., 2021) and although methods have demonstrated reliable estimations, they provide little to no lead time for redirection of patients or reallocation of resources (Locey et al., 2020; Nguyen et al., 2021). Therefore, a methodology that could provide advanced warning of the COVID-19 hospitalization burden would be advantageous such as through wastewater surveillance.

Wastewater surveillance (routinely testing wastewater for public health benefit) has been utilized to understand the epidemiology of various pathogens, such as poliovirus and norovirus, for over 40 years (Sinclair et al., 2008). Biological agents, including the aforementioned pathogens, enter the sewer system through various excrements such as feces, urine, and saliva (McClary-Gutierrez et al., 2021). SARS-CoV-2 RNA is shed in the feces of symptomatic and asymptomatic infected individuals allowing use of wastewater surveillance to estimate COVID-19 transmission across entire communities (Daughton, 2020). Wastewater surveillance provides low-cost, high coverage, non-invasive, and timely estimates of COVID-19 transmission for large populations and is not reliant on individuals seeking, obtaining, and reporting laboratory-based diagnostic testing (Larsen & Wigginton, 2020). The ability to monitor reappearances, surges, and transmission risks in communities has resulted in many health agencies adopting wastewater surveillance to complement their existing measures for tracking COVID-19 (Shah et al., 2022).

Early in the pandemic, wastewater surveillance was shown to be a leading indicator of COVID-19 cases and hospitalizations (Fernandez-Cassi et al., 2021). Predictive models for cases and hospitalizations have used compartmental modelling approaches with wastewater as a predictor (Nourbakhsh et al., 2022), while others have used time-series approaches (Schenk et al., 2023). Wastewater data was found to systematically improve predictive models of COVID-19 cases at the community-level in Europe (López-Peñalver et al., 2023; Wang et al., 2022), and the U.S. (Jeng et al., 2023) and, with the underreporting of case data, wastewater surveillance is likely to improve hospitalization forecasting (Peccia et al., 2020). Therefore, we argue that a predictive model incorporating wastewater for hospitalization outcomes would be highly useful for predictions of disease transmission to improve resource distribution and surge preparation. Furthermore, a predictive model of COVID-19 related hospitalizations that has advanced lead time granted by wastewater surveillance could alleviate stress on the healthcare system and ensure proper medical resource allocation for localized management. Herein, we model COVID-19 related hospitalizations from wastewater throughout New York State (NYS) at different geographic scales, and we improve upon models that only use clinical case data. Further, we evaluate the benefit of models that use wastewater against those using clinical data. In addition, we evaluate the quality of predictions based on models built using historic data versus recent data and the changing impact of key variables including case data, population vaccinated, and wastewater.

## 2 Methods

### 2.1 Setting and context

Wastewater surveillance of COVID-19 began in NYS in April of 2020. Various universities and local health departments partnered with wastewater treatment plants (WWTPs) to test wastewater for SARS-CoV-2. Beginning in January of 2021, wastewater surveillance began scaling to all counties throughout the state (Neyra et al., 2023). Our analysis herein includes data from 109 WWTPs across 56 counties in NYS covering over 13.8 million people, and we included data through June 30, 2022.

### 2.2 Data

#### 2.2.1 Wastewater data

Wastewater samples were collected from each site across NYS using twenty-four hour (24 h) composite sampling. Samples were then shipped to regional laboratories for extraction and quantification of SARS-CoV-2 virus. Each regional lab used a different processing method before quantifying viral-RNA. Briefly, NYC processed samples using centrifugation prior to virus concentration using polyethylene glycol (PEG) precipitation and quantified results of 24-h (24 h) composite samples using reverse transcription quantitative polymerase chain reaction (RT-qPCR) of the SARS-CoV-2 N1 gene (Hoar et al., 2022). Quadrant Biosciences analyzed most of Upstate NY counties processing samples using ultracentrifugation through a sucrose cushion and quantifying concentrations of SARS-CoV-2 IP2/IP4 genes using RT-qPCR (Wilder et al., 2021). The State University of New York at Stony Brook (Stony Brook) processed 24 h composite samples for Suffolk County using centrifugation followed by PEG precipitation and quantified concentrations of SARS-CoV-2 N1 gene using digital droplet PCR (DD-PCR). Lastly, The State University of New York at Buffalo (UB-SUNY) analyzed data for the Western New York Region using two methods during the study period. Method one (UB-SUNY 1) processed samples using electronegative membrane filtration and quantified concentrations using RT-qPCR for samples collected between May 2020 and April 17, 2022. Beginning April 18, 2022, method two (UB-SUNY 2) processed samples using Nanotrap*®* Microbiome A Particles (previously called Nanotrap® Magnetic Virus Particles; Ceres Nanoscineces) and quantified SARS-CoV-2 N2 gene using RT-qPCR. Different lab methods were included in the analysis models as fixed effects. The sensitivity of Quadrant Biosciences method (the lab with most of the state’s surveillance sites) was 1 COVID-19 case in 10,000 population and the limit of quantification was 5 gene copies per milliliter (Wilder et al., 2021). Appendix A provides additional details on laboratory methods.

#### 2.2.2 Clinical data

We obtained COVID-19 hospitalization data for the period January 2020–June 2022 from the NYS DOH Statewide Planning and Research Cooperative System (SPARCS). Patient data were geocoded to the sewershed and county levels and aggregated into counts of new COVID-19 hospital admissions per day. See Appendix A for more details on geocoding methods.

Hospital admissions were restricted to in-patient and adjusted to be per 100,000 population estimates (Hill & Larsen, 2023). We excluded individuals testing positive for COVID-19 after arrival to the hospital for unrelated reasons including out-patient visits, emergency visits not admitted to the hospital, and patients tested prior to surgery or hospitalization unrelated to COVID-19. We also obtained county-level daily vaccination coverage from NYS DOH (DOH, 2022b). Hospital admissions were our primary outcome variable, but we also include hospital admissions on the day of the wastewater sample in our models for two reasons. First, this variable acts as an intercept for the model providing the starting point for each prediction. Second, it allowed us to compare the predictive ability of wastewater against the predictive ability of the hospitalization trend to determine how much new information wastewater can add to forecasting models for hospital admissions.

#### 2.2.3 Demographic and social vulnerability data

We obtained sewershed and county level population demographic variables for age and population density from the U.S. Census 2019 American Community Survey using methods previously described (Hill & Larsen, 2023). We apportioned the block group data to sewersheds then aggregated to the sewershed level to get estimates for the following measures: proportion of people over fifty years old, proportion of males over fifty, and population density. These covariates were selected because they helped control for population dynamics of disease spread (i.e., age is a comorbidity, and population density is associated with infection transmission dynamics). We obtained the census-tract level social vulnerability index (Flanagan et al., 2011) from the U.S. Centers for Disease Control (CDC) and calculated the mean, standard deviation, and Gini coefficient for census tracts intersecting sewersheds.

#### 2.2.4 Health covariates

We considered county-level estimates of the following comorbidities known to increase the risk of COVID-19 hospitalization: cancer from the state cancer registry (DOH, 2022a), asthma and respiratory disease rates as well as a combined indicator for cirrhosis, diabetes, and kidney (CDK) disease rates from Community Health Indicator Reports (New York State Community Health Indicator Reports (CHIRS), 2022), and obesity from the New York State Expanded Behavioral Risk Factor Surveillance System (CDC, 2022). Each of these covariates were selected because they are comorbidities for COVID-19.

#### 2.2.5 Data processing

All time-series data were converted to 7-day right-adjusted rolling daily averages (wastewater, cases, test positivity, hospital admissions). The 7-day average accounted for weekend effects of treatment seeking behavior. A shorter time-interval was not selected because most wastewater sampling sites only sampled once per week. All covariate data were county or sewershed level and did not change over time. There were no missing covariate data. For our time-series data, wastewater data were included for all sites when available. Time gaps of greater than eight days were removed from the model since 7-day rolling averages could not be calculated for those. Clinical case data were complete for all days for all counties. Hospitals admissions that were missing for certain county-day combinations were replaced with zero since they were days with no admissions reported (DOH, personal communication).

### 2.3 Sensitivity and specificity of wastewater trends

We first compared trends in SARS-CoV-2 RNA concentrations in wastewater to trends in new hospital admissions over rolling 14-day intervals. The trend direction was measured using the linear coefficient for the 14-day interval for wastewater and hospitalizations. Increases were classified as positive coefficients and decreases as negative coefficients. We used three thresholds to test the sensitivity of wastewater correctly classifying an increase of greater/less than 0%, greater/less than 5%, and greater/less than 10%. We calculated the sensitivity (proportion of increasing trends in hospitalizations identified by increasing trends in wastewater), specificity (proportion of decreasing trends in hospitalizations identified by decreasing trends in wastewater), positive predictive value (PPV) (the probability that an increasing trend in the wastewater accurately indicated an increasing trend in hospitalizations) and negative predictive value (NPV) (the probability that a decreasing trend in wastewater accurately indicated a decreasing trend in hospitalizations) across the state. We used a 10-day lag of the values for the trend because 10-days was the optimal lag for correlation.

### 2.4 Model specification for hospitalizations

We modeled 7-day average hospitalizations per 100,000 population as a function of the lagged 7-day average amount of SARS-CoV-2 RNA in wastewater using a generalized linear mixed model (GLMM) approach specified in Equation (1):

1 $$y_{i_{c}}∼Poisson\left(e^{x_{i_{c}}\beta_{i_{c}}}+\lambda_{c}\right)$$

Equation (1): Where $y_{i}$ is the number of new hospital admissions for each observation for each county denoted as c, $x_{i_{c}}$ is a vector of predictors, and $\beta_{i_{c}}$ is a vector of coefficients. $\lambda_{c}$ is a vector of random intercepts for each county. We also repeated this model at the sewershed level.

The GLMM model was selected because our count data had a Poisson distribution and included spatially nested repeated measures that could be analyzed with a mixed-model component through a random intercept. The 7-day average was calculated by first taking the linear approximation of SARS-CoV-2 RNA concentration levels per sampling site between sample points. This provided daily estimates for each location since sampling frequency of wastewater varied between once weekly and three times weekly. Data were then aggregated to the county, regional, and state levels using a population weight for each site within the jurisdiction.

Hospitalization data best fit a Poisson distribution, after checking for overdispersion with both a negative binomial and zero-inflated Poisson approach. We improved the fit of the model by incorporating the 10-day lagged 7-day average test-positivity as a covariate and tested additional covariates using a stepwise approach comparing model Akaike information criteria (AIC) and eliminating variables that did not contribute to improving the model fit. Variables remained even if insignificant if their effect was in the hypothesized direction (e.g., comorbidity for CDK rate was positive but not significant so it remained, asthma rate was negative but not significant so it was removed). We selected the optimal lag between wastewater and hospitalizations of 10 days by comparing Pearson correlation values for lags of 0–30 days.

We modeled hospitalizations at four separate geographic scales: the state level, the regional level, the county level, and at the WWTP sewershed. We included a random intercept for each geographic scale with the sewershed model having a random intercept for sewershed, the county model having a random intercept for county, and the regional model having an intercept for region. We focused most of our analyses at the county level, including more detailed model exploration, because most public health decisions are made at the county level or above (groups of counties forming each region).

Wastewater data being community samples, and potentially influenced by environmental factors, are often adjusted into viral load normalized values using wastewater flow, human-fecal, or chemical measures quantified in the sample (Hoar et al., 2022; Wilder et al., 2021). Limitations in data availability for each lab and region made use of a single normalizing value difficult (e.g., some sites had no flow data, some labs did not measure a human-fecal indicator), however, for within lab correlations comparisons, we evaluated whether normalizing the data improved correlations with clinical data. The natural log of raw gene copies correlated with case and hospitalization data more than any other normalization such as viral copies per WWTP flow or copies per fecal indicator. While used less often, the raw data across the state had the strongest associations with the outcome and we determined not to normalize the data beyond log-transformation. Wastewater data averaged over rolling 7-day intervals lagged 10 days correlated best with hospital admissions. We did not detect any temporal or spatial autocorrelation in the model residuals and models with autoregressive structures to account for time-series correlation did not improve model fit and were thus not extensively explored.

### 2.5 Model accuracy evaluation and predictive ability

We originally fit a model from April 29, 2020 to March 12, 2022 and tested the model using two sets of data. The first was from the same time period but randomly left out records from the original model (80% of data used in the model for training, 20% excluded and used for testing). A second out-of-data validation dataset was created using data from March 12, 2022 to June 1, 2022. This model had good predictive ability for the in-data predictions (Pearson correlation of observed v. predicted with r = 0.89), but poor predictive ability for the out-of-data predictions (r = 0.33), which led us to evaluate if any of our covariates' predictive ability changed over time. To do this, we fit a second model that only used data from March 1, 2022 to June 30, 2022 and compared this to the model results from the original model. We also fit a third model using data for all time and compared the scaled coefficients across the models to see how their predictive ability changed. Evaluation of the models was done using two datasets. The first using in-data observations (20% of the recent data randomly left out of the model fitting process). The second validation dataset used out-of-data predictions and was made up of five counties excluded from the training data plus data from June 1, 2022 to June 30, 2022. This dataset provided outside time points and outside sample collection sites. We compared the predicted values to the observed values using the mean absolute scaled error (MASE). The MASE is a numeric value that allows for comparison between models with different structure and data with lower MASE values indicating lower error in the predictions.

## 3 Results

### 3.1 Statewide hospital admissions

**Fig. 1:**
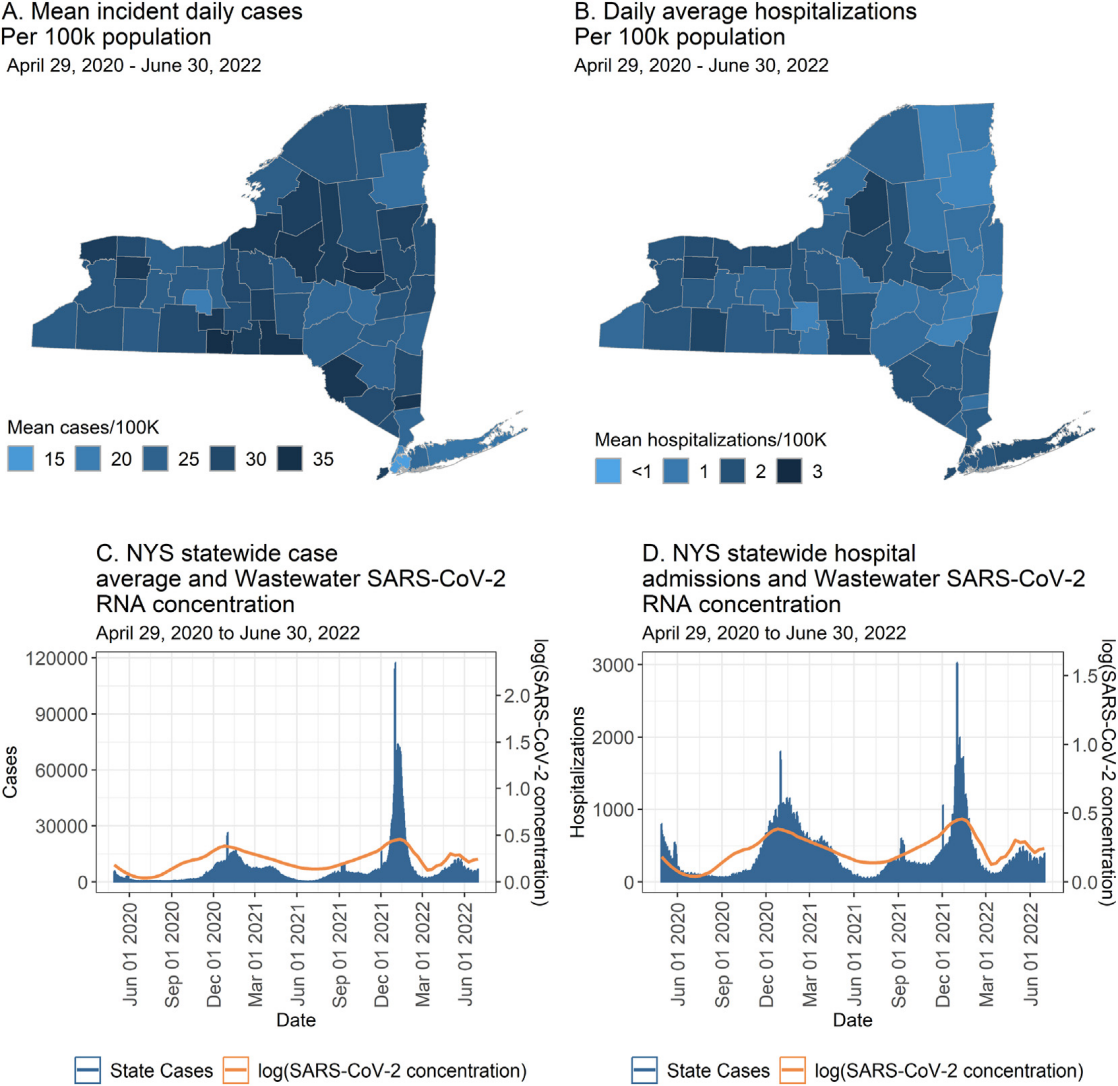
A) Average positive test rate for new cases per 100,000 population. B) Average daily hospital admissions per 100,000 population. C) Total incident COVID-19 cases in NYS and the mean trend of wastewater results. D) Total new daily COVID-19 hospital admissions over time and mean trend of wastewater results.

**Fig. 2:**
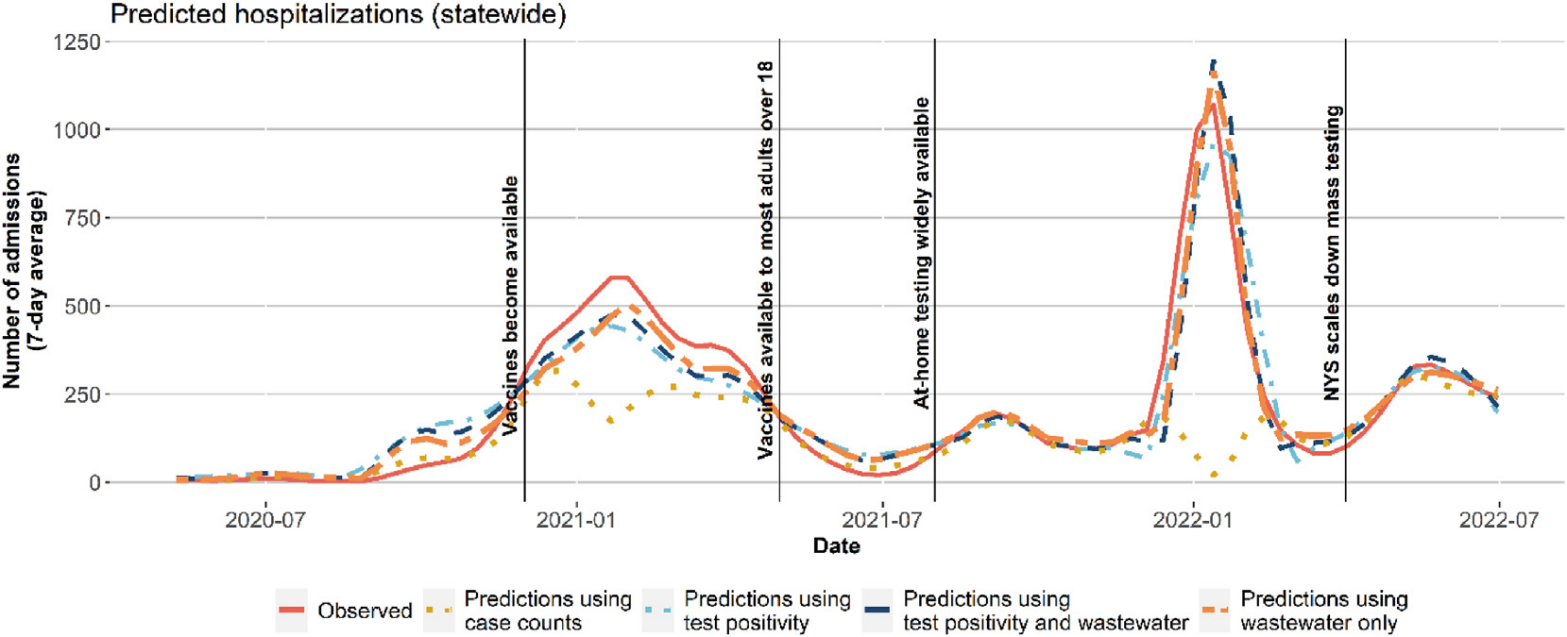
Statewide prediction (smoothed average) of new hospital admissions for models using covariates and case counts, covariates and test positivity, covariates, test positivity and wastewater, and predictions from the model with covariates and wastewater only.

From April 29, 2020 through June 30, 2022, hospital admissions varied across NYS with some upstate counties seeing high admissions per 100,000 population but generally low admissions overall in absolute numbers (Fig. 1a and b). Wastewater detection levels generally follow the rise and decline of cases and hospitalizations across NY (Fig. 1c and d) In addition, new hospital admissions were highest at the start of the pandemic and during the Delta wave (Fall 2021) and surges of 2020–2021 and 2021–2022 (Fig. 2).

### 3.2 Sensitivity and specificity of wastewater trends

We found that two-week trends in wastewater results correlated with trends in hospital admissions of COVID-19. Overall, sensitivity of classification of hospital trends from wastewater was 56% (95% CI [0.507, 0.613]). PPV and specificity increased as the magnitude of the change in trend increased with a PPV of 58% (95% CI [0.511, 0.654]) when the threshold of change was at 10%. Specificity was highest for wastewater predicting the trend for hospital admissions when the threshold was at 10% with a specificity of 81% (95% CI [0.76, 0.85]).

### 3.3 Forecasting hospitalizations

Correlation between 7-day average of SARS-CoV-2 wastewater results lagged 10 days and the 7-day average of hospital admissions varied by laboratory with a statewide Pearson correlation of 0.415 (p < 0.001). UB-SUNY 1 had the highest correlation of 0.73 with NYC close behind (r = 0.72) and Stony Brook with correlation of 0.69. Quadrant and UB-SUNY 2 had the lowest correlations of 0.44 and 0.19 respectively, but both were statistically significant (p < 0.001).

Wastewater surveillance data improved hospitalization forecasting (Fig. 2). Case data had good correlation with new hospital admissions in 2020 but worsening predictive ability during surges and after at-home testing expanded in August 2021 (Fig. 2). Case data model prediction was off by a mean of 0.09 per 100,000 population with a lower limit of −16.19 and upper limit of 8.32. Compared to test positivity with an average difference of 0.02 (range = [−3.3, 7.29]) and wastewater with average difference of 0.01 (range = [−1.95, 2.99]), meaning cases were inferior as a predictor. Models with wastewater and test positivity separately, along with vaccination and covariate data, performed well at predicting new hospital admissions. The best performing models were those that included both wastewater and test positivity.

Wastewater significantly improved model predictions at the state level with an increase in MASE of 11.2% (Table 1). Wastewater improved the predictive ability of each geographic scale increasing the regional model’s accuracy by 14.63%, increasing the county model’s accuracy by 10.57%, and increasing the sewershed model’s accuracy by 4.69% from models using covariates and test positivity only (Table 1). In addition, wastewater was a consistently important predictor in each model and was the most important predictor in the regional model (β = 0.33, SD = 0.03, p < 0.0001, Table 1) and county level model (β = 0.38, SD = 0.01, p < 0.0001, Table 1) compared to other predictors. SVI was only significant in the sewershed model with sewersheds having higher SVI associated with greater rates of hospitalization (β = 25, SD = 0.04, p < 0.0001).

**Table 1:** Model estimates (scaled) for all geographies. Models were first fit with test positivity only and covariates, then fit with test positivity, wastewater, and covariates to compare the change in accuracy with the addition of wastewater. Models fit using data from April 29, 2020 to March 12, 2022.

	State		Region		County		Sewershed	
	*With test positivity*	*With test positivity and wastewater*	*With test positivity*	*With test positivity and wastewater*	*With test positivity model*	*With test Positivity and wastewater*	*With test positivity*	*With test positivity and wastewater*
Intercept	**5.46 (1.6) p** = **0.00066**	**5.57 (0.43) p** < **0.0001**	0.65 (0.35) p = 0.06	**0.73 (0.28) p** = **0.01**	**0.69 (0.33) p** = **0.036**	**0.83 (0.34) p** = **0.015**	**0.9 (0.43) p** = **0.036**	0.27 (0.6) p = 0.65
Hospital admissions per 100 k on sample day	−0.06 (0.08) p = 0.43	−0.13 (0.08) p = 0.094	**0.11 (0.02) p** < **0.0001**	**0.11 (0.02) p** < **0.0001**	**0.12 (0.01) p** < **0.0001**	**0.12 (0.01) p** < **0.0001**	**0.15 (0.01) p** < **0.0001**	**0.17 (0.02) p** < **0.0001**
Quadrant^a^	–	–	−0.09 (0.37) p = 0.81	−0.32 (0.3) p = 0.29	−0.3 (0.42) p = 0.47	−0.61 (0.43) p = 0.16	0.08 (0.49) p = 0.87	0.53 (0.72) p = 0.46
Stony Brook^a^	–	–	0.12 (0.48) p = 0.81	−0.23 (0.38) p = 0.55	0.21 (0.75) p = 0.78	−0.32 (0.77) p = 0.68	−0.15 (0.87) p = 0.87	−0.65 (1.12) p = 0.57
UB-SUNY 1^a^	–	–	0.15 (0.48) p = 0.76	0.16 (0.39) p = 0.68	0.18 (0.73) p = 0.81	0.08 (0.75) p = 0.92	0.1 (0.89) p = 0.91	0.3 (1.16) p = 0.8
CDK^b^ rate	–	–	–	–	0.14 (0.16) p = 0.37	0.14 (0.16) p = 0.4	0.01 (0.13) p = 0.94	0.02 (0.19) p = 0.9
BMI 30th percentile rate	–	–	–	–	−0.02 (0.13) p = 0.91	0.05 (0.14) p = 0.72	0.27 (0.17) p = 0.12	−0.1 (0.32) p = 0.76
SVI	–	–	–	–	0.14 (0.14) p = 0.29	0.11 (0.14) p = 0.42	**0.06 (0.01) p** < **0.0001**	**0.25 (0.04) p** < **0.0001**
Percent of population over 50 years old	–	–	–	–	0.06 (0.12) p = 0.61	0.05 (0.13) p = 0.68	**0.28 (0.01) p** < **0.0001**	**0.43 (0.04) p** < **0.0001**
Minor holiday^c^	−0.01 (0.02) p = 0.44	−0.03 (0.02) p = 0.11	**−0.25 (0.09) p** = **0.0068**	**−0.18 (0.09) p** = **0.049**	**−0.09 (0.03) p** = **0.008**	**−0.1 (0.03) p** = **0.0035**	−0.04 (0.04) p = 0.24	0.06 (0.11) p = 0.55
Not a holiday^c^	−0.02 (0.02) p = 0.22	−0.03 (0.02) p = 0.083	**−0.39 (0.06) p** < **0.0001**	**−0.28 (0.06) p** < **0.0001**	**−0.3 (0.02) p** < **0.0001**	**−0.26 (0.02) p** < **0.0001**	**−0.17 (0.03) p** < **0.0001**	−0.11 (0.08) p = 0.16
Percent of population with two doses of mRNA vaccine	**−0.81 (0.2) p** < **0.0001**	**−0.68 (0.18) p** = **0.0002**	**−0.18 (0.03) p** < **0.0001**	**−0.16 (0.03) p** < **0.0001**	**−0.27 (0.01) p** < **0.0001**	**−0.22 (0.01) p** < **0.0001**	**−0.21 (0.01) p** < **0.0001**	−0.1 (0.06) p = 0.13
Ln (test positivity)	**0.32 (0.07) p** < **0.0001**	**0.31 (0.07) p** < **0.0001**	**0.46 (0.02) p** < **0.0001**	**0.30 (0.03) p** < **0.0001**	**0.48 (0.01) p** < **0.0001**	**0.29 (0.01) p** < **0.0001**	**0.31 (0.01) p** < **0.0001**	**0.19 (0.03) p** < **0.0001**
Ln (SARS-CoV-2 raw gene copies)	–	**0.14 (0.03) p** < **0.0001**	–	**0.33 (0.03) p** < **0.0001**	–	**0.38 (0.01) p** < **0.0001**	–	**0.14 (0.04) 0.00023**
n	370	370	1022	1022	6281	6281	3228	491
AIC	3302.14	3287.64	2815.189	2719.519	17835.747	17124.39	18408.021	1843.959
MASE	15.25	13.54	1.23	1.05	1.23	1.1	0.64	0.61
Change in MASE when wastewater added	+11.2%		+14.63%		+10.57%		+4.69%	
Conditional Nakagawa R^2^ (fixed and random effects)	–	–	0.44	0.48	0.58	0.63	0.74	0.8
Marginal Nakagawa R^2^ (fixed effects only)	0.07	0.19	0.32	0.4	0.3	0.36	0.22	0.27

*Note:* Estimate (SD), p value. ^a^Reference group is NYC, ^b^ Cirrhosis, diabetes, and kidney disease, ^c^Reference group is major holiday.

Other variables tested that were removed due to insignificant contribution and wrong direction from expected were: cancer rate, respiratory disease rate, asthma rate, and population density.

A one standard deviation change in natural log-transformed raw copies of SARS-CoV-2 in wastewater led to a 9% increase in hospitalizations ten days later (p < 0.0001, Table 2). Test positivity was also positively associated with an increase in new hospital admissions with a one standard deviation increase in test positivity leading to a 42% increase in hospitalizations ten days later (p < 0.0001). A one-standard deviation increase in COVID-19 vaccination coverage led to a 78% decrease in hospitalizations (p = 0.0072). Increasing rate of CDK disease was also associated with increasing hospitalizations (p = 0.026).

**Table 2:** County model fit for data collected between March 1, 2022 and June 1, 2022 and validated using data from June 1, 2022 to June 30, 2022. Predictor variables are scaled and the outcome (new COVID-19 hospital admissions per 100 k population) is on its original scale.

	With covariates	With covariates and vaccination	With covariates, vaccination, and wastewater	With covariates vaccination, and test positivity	With covariates, vaccination, test positivity, and wastewater
Intercept	−0.36 (0.31) p = 0.26	−0.29 (0.31) p = 0.35	−0.02 (0.32) p = 0.95	0.05 (0.3) p = 0.86	0.14 (0.3) p = 0.63
Hospital admissions per 100 k on sample day	**0.36 (0.02) p** = < **0.0001**	**0.37 (0.02) p** < **0.0001**	**0.32 (0.02) p** < **0.0001**	**0.18 (0.03) p** < **0.0001**	**0.17 (0.03) p** < **0.0001**
Quadrant^a^	**0.72 (0.34) p** = **0.036**	0.64 (0.33) p = 0.054	0.32 (0.35) p = 0.36	0.04 (0.32) p = 0.9	−0.06 (0.33) p = 0.86
Stony Brook^a^	−0.35 (0.58) p = 0.54	−0.43 (0.56) p = 0.44	−0.71 (0.58) p = 0.22	−0.42 (0.54) p = 0.43	−0.55 (0.55) p = 0.32
UB-SUNY 1^a^	0.13 (0.42) p = 0.76	0.04 (0.41) p = 0.92	0.06 (0.42) p = 0.89	−0.2 (0.4) p = 0.61	−0.17 (0.4) p = 0.67
UB-SUNY 2^a^	0.8 (0.41) p = 0.051	0.72 (0.4) p = 0.071	0.25 (0.41) p = 0.54	−0.29 (0.39) p = 0.45	−0.42 (0.39) p = 0.28
CDK^b^ rate	**0.18 (0.09) p** = **0.049**	**0.19 (0.09) p** = **0.033**	**0.21 (0.09) p** = **0.025**	**0.19 (0.09) p** = **0.032**	**0.2 (0.09) p** = **0.026**
BMI 30th percentile rate	0.08 (0.09) p = 0.37	0.04 (0.09) p = 0.63	0.05 (0.09) p = 0.59	−0.06 (0.09) p = 0.52	−0.04 (0.09) p = 0.61
SVI	−0.07 (0.09) p = 0.43	−0.1 (0.09) p = 0.25	−0.13 (0.09) p = 0.17	−0.1 (0.09) p = 0.27	−0.11 (0.09) p = 0.23
Percent of population over 50 years old	**0.24 (0.09) p** = **0.0068**	**0.23 (0.08) p** = **0.0049**	**0.23 (0.09) p** = **0.0072**	0.15 (0.08) p = 0.061	0.16 (0.08) p = 0.054
Minor holiday^c^	**−0.28 (0.05) p** < **0.0001**	**−0.28 (0.05) p** < **0.0001**	**−0.23 (0.05) p** < **0.0001**	0.01 (0.05) p = 0.85	1.1 (0.05)
					1.2 p = 0.91
Not a holiday^c^	**−0.08 (0.04) p** = **0.029**	**−0.08 (0.04) p** = **0.029**	**−0.08 (0.04) p** = **0.031**	0.05 (0.04) p = 0.17	1.3 (0.04)
					1.4 p = 0.27
Percent of population with two doses of mRNA vaccine	–	−0.1 (0.09) p = 0.27	−0.13 (0.09) p = 0.16	**−0.23 (0.09) p** = **0.0079**	**−0.24 (0.09) p** = **0.0072**
Ln (test positivity)	–	–	–	**0.38 (0.02) p** < **0.0001**	**0.35 (0.02) p** < **0.0001**
Ln (SARS-CoV-2 raw gene copies)	–	–	**0.19 (0.02) p** = < **0.0001**	–	**0.09 (0.02) p** = < **0.0001**
n	3879	3879	3879	3879	3879
AIC	9015.612	9016.495	8926.354	8711.753	8697.352
MASE	0.75	0.75	0.67	0.57	0.53
Change in MASE	–	0%	+10.67%	+14.93%	+7.02%
Mean difference and 95%CI	0.14 (0.13, 0.16)	0.14 (0.12,0.15)	0.072 (0.059,0.086)	0.015 (0003,-0.029)	0.013 (0.0022,0.025)
Conditional Nakagawa R^2^ (fixed and random effects)	0.39	0.41	0.42	0.42	0.43
Marginal Nakagawa R^2^ (fixed effects only)	0.19	0.19	0.21	0.24	0.25

*Note:* Estimate (SD), p value. ^a^Reference group is NYC, ^b^cirrhosis, diabetes, and kidney indicators ^c^Reference group is major holiday. Other variables tested that were removed due to insignificant contribution and wrong direction from expected were: cancer rate, respiratory disease rate, asthma rate, and population density.

**Fig. 3:**
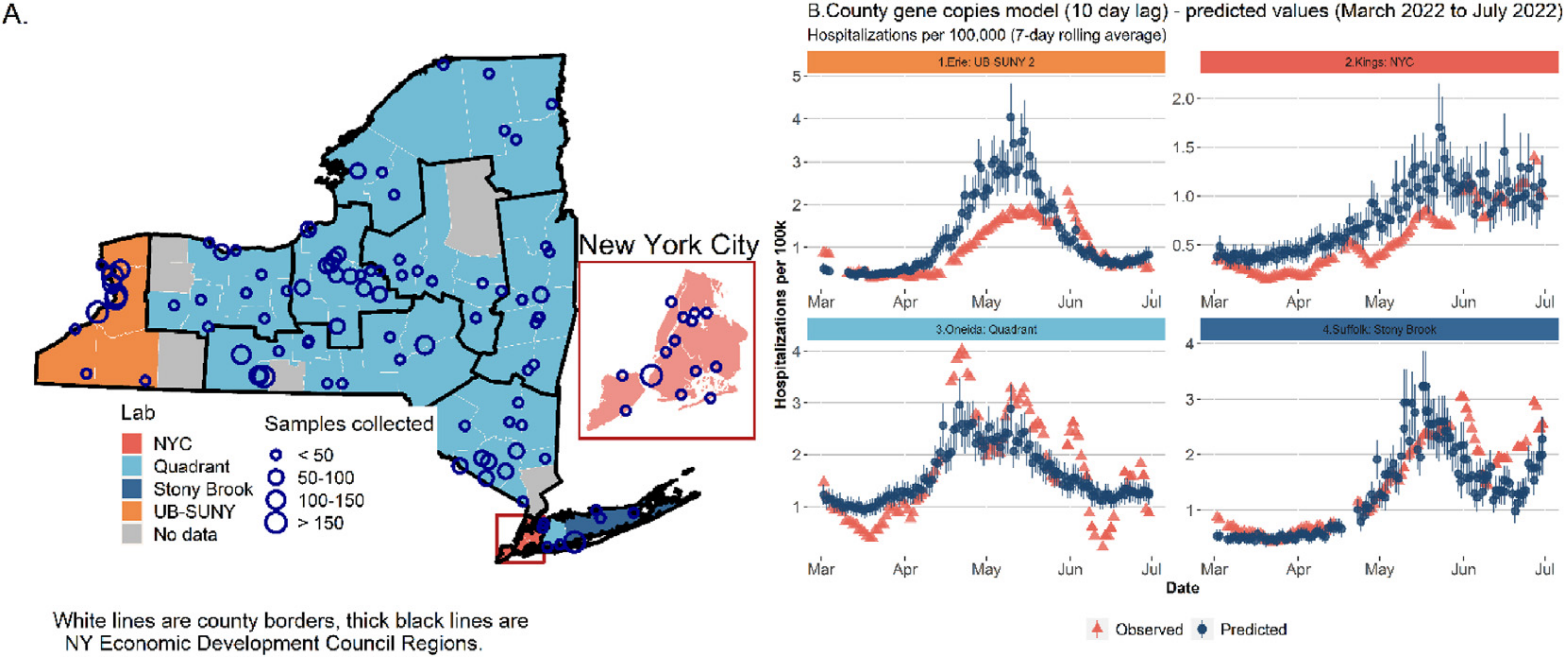
A: Four laboratories analyzed wastewater data across NY using different methods. In total, 109 WWTPs data were incorporated in this study serving 13.8 million people. B1: The UB-SUNY lab’s data for Erie county had good predictive fit with moderate overprediction during the summer of 2022. B2: Kings County analyzed by NYC had very good model fit but wider prediction intervals. B3: Quadrant analyzed most of New York’s data and had good fit in Oneida county. B4: Stony Brook University analyzed data for Suffolk County on Long Island and their predicted data also had good fit to the observed.

Forecasts proved very stable across labs despite different methods (Fig. 3b).

Wastewater significantly improved forecasting county hospitalizations, improving the model with an increase in MASE of 7.02% (Table 2) beyond the model without wastewater. In addition, wastewater significantly reduced the average difference between predicted and observed values. The model with wastewater and test positivity had a mean difference in the predicted new hospital admissions of 0.01 per 100,000 population (95% CI [0.002, 0.025], IQR [-0.227,0.215]).

The models' accuracy at predicting the testing dataset was generally good with a correlation between predicted and observed values of 0.7689 for in-data predictions. Out-of-data predictions were less accurate but still highly correlated with observed new hospital admissions (r = 0.5585).

**Fig. 4:**
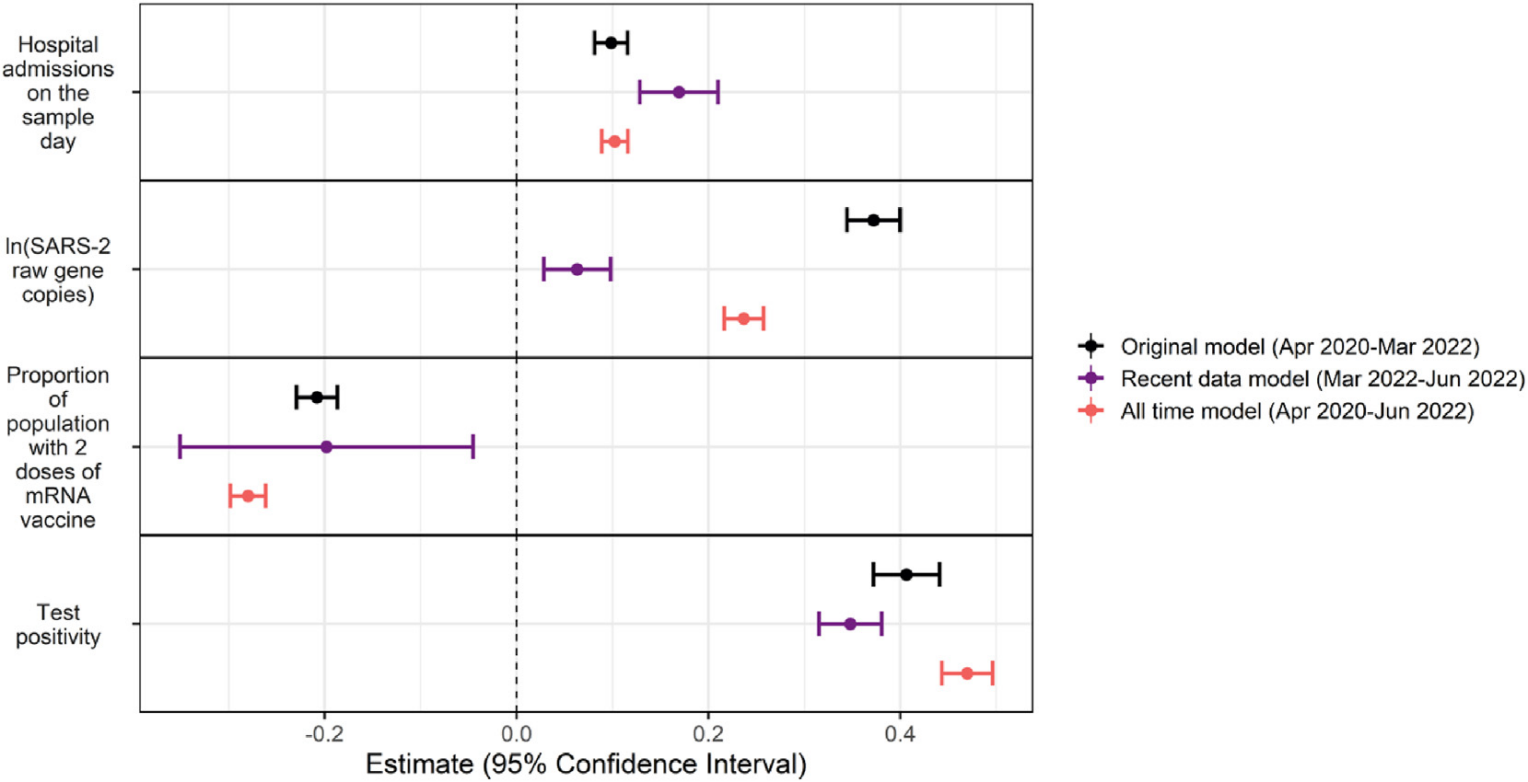
Comparison of model coefficients for Model 1: fit with data between April 29, 2020 and March 12, 2022; Model 2: fit with data between March 1, 2022 and June 30, 2022; and Model 3: fit with data over all time (April 29, 2020 to June 30, 2022). Estimates are scaled and centered.

Model predictions were found to be the highest for the recent data model (data from 2022 only). The original model fit using data from 2020 to 2021 did not predict 2022 data very well with a correlation coefficient of 0.33 between the observed and predicted values. The fitting of a model using recent data only (March 2022 to June 2022) resulted in more accurate predictions. Comparing the standardized coefficients for the original model, the recent data model, and a model for all-time showed that the effect size of some covariates and uncertainty around the effect sizes changed (Fig. 4). Specifically, the wastewater variable decreased in its effect size, though the error around the estimate remained similar (Fig. 4). Test positivity was robust to the changing time period with not much change in effect size or error (Fig. 4). Vaccination status, however, changed significantly in the error around the effect. The interval size increased from (−0.22, −0.18) to (−0.35, −0.05), which is a more than seven-fold increase in the error around that estimate. Thus, the recent data model was selected for making predictions since it is most relevant going forward for predicting future hospitalization outcomes.

## 4 Discussion

### 4.1 Wastewater as a predictor of new COVID-19 hospital admissions

We found wastewater to greatly improve near-term (ten-day) hospitalization forecasting. Trends in wastewater showed moderate sensitivity in detecting trends in incident COVID-19 hospital admissions, but the real power in wastewater was the added value in forecasting hospitalizations. The amount of SARS-CoV-2 RNA found in wastewater accurately predicted new COVID-19 hospitalizations ten days in advance with more accuracy than COVID-19 test positivity or incidence.

These results indicate greater lead time for wastewater surveillance as a predictor of hospitalizations than clinical surveillance. The ten-day lead time is in line with findings from a previous study from Athens, Greece (Galani et al., 2022). In addition, the ten-day lead time is an important finding for public health officials because it can help with planning for predicted surges in new admissions and coordinate response efforts. In addition, the ten-day lead time is a product of quantifiable levels increasing in wastewater prior to the onset of symptoms in infected individuals (Larsen et al., 2022) plus the time it may take for an individual to become sick enough that they need hospital admission. Advance notice can allow communities to respond accordingly and provides another data source of public health officials tracking the disease. This lead time is also important from a modelling and methods perspective to ensure that the optimal fit for a model is achieved to maximize predictive power of the model and to understand why wastewater data might not peak at the same time as new hospital admissions in a community (Olesen et al., 2021). These models share the ability of other published models of forecasting peaks in new COVID-19 hospital admissions (Fernandez-Cassi et al., 2021) with the added ability to make accurate predictions during times of lower transmission. The value of forecasting the decline of hospitalizations during a surge should not be underappreciated. These results also show the ability to predict new COVID-19 hospital admissions at multiple spatial aggregations (from the state level on down to the sampling point level). We found important limitations for variables such as community vaccination coverage that might indicate varying levels of immunity during different times. While two doses of mRNA vaccine were a significant predictor of fewer new hospital admissions across the entire time period, the error around the effect size has dramatically increased over time suggesting that modelling hospitalizations would benefit from more dynamic estimates of the population’s immunity status. Further, our findings that the model using the most recent data was the best model could be indicative of many factors including changes in case reporting over time (e.g., introduction of at-home tests), changes in immune status due to previous infection, and changes in the virulence of the virus based on new variants competing for dominance. All or some of these might influence local and near-term conditions of the virus potentially indicating that models built more dynamically using recent data might be more predictive. This is worth future investigation to compare models with varying window sizes for recent data to determine if forecasting can be improved by limiting the inclusion of older data but also to learn if models can effectively predict increases from periods of low transmission.

The accuracy of statewide predictions in these analyses provide several public health benefits. First, wastewater surveillance for public health benefit does not require intense sampling frequencies – the majority of the data used here was from weekly sampling. Weekly sampling is much less burdensome on wastewater treatment plant operators who actually pull the sample. Second, our statewide models combined wastewater from different laboratories all using unique methods to quantify the amount of SARS-CoV-2 in wastewater. These results suggest that programs across jurisdictions might see benefit in combining data even if the methods differ. Third, the statewide estimate provides a concise message for policymakers. Aggregating community-level surveillance metrics into a statewide estimates resulted in accurate models and could benefit the decision-making process at larger geographic scales.

### 4.2 Limitations

A number of limitations are present in this study. First, the address of a hospitalized individual may not align with the location that individual contracted COVID-19 and the hospital admissions data excludes anyone hospitalized in NYS with an address elsewhere. We expect these numbers to be small and their influence on the results to be minimal. Second, a number of covariates we selected as potential predictors of hospitalizations were not available at the sewershed level. Vaccination and comorbidity data within sewersheds might improve the predictive ability of the sewershed-level model. Further, vaccination and testing rates vary based on access to healthcare, a factor not specifically accounted for in our model. Also, while our model integrated data from multiple labs with a fixed effect, the between-lab variance might be masking some correlations and effects that are stronger with single-lab analyses, such as we observed with single-lab correlations being stronger than the overall correlations.

In addition, we used log-transformed raw gene copies of SARS-CoV-2 per site without a normalizing method like viral copies per flow, which is a less traditional approach. Normalizing wastewater data is often recommended to improve the signal from quantification results (Hsu et al., 2022), however, there are also studies that have shown that normalization gives comparable results to non-normalized data (Rainey et al., 2023). Further, flow is considered an excellent normalizing variable (Rainey et al., 2023), however, flow data was completely missing for the majority of sites across NYS particularly for smaller sampling sites. Imputation might be possible but would require another data source. Thus, while using the raw data might have introduced some noise in the analysis, we did not detect any impediment to the model predictions from using raw data. The use of the raw data also allowed for inclusion of data from each lab despite differences in extraction methods and lack of available normalizers.

### 4.3 Implications for public health policy

As mass COVID-19 testing sites close and more COVID-19 tests are conducted at home (Shah et al., 2022), data from wastewater surveillance will become even more valuable to understand COVID-19 transmission and guide the public health response. These results suggest that wastewater surveillance can increase the accuracy of predictive models built using clinical data by as much as 15%. Given the relatively low cost of wastewater surveillance ($225.00 per sample, with shipping included, in NYS), large geographic areas can be surveilled quickly and consistently avoiding selection bias that might come from clinical testing. Further, NYS wastewater data are timely with 80% of samples reported within two days and 90% within three days (Neyra et al., 2023) meaning that most sites will get seven to eight days of lead time for potential increases in new hospital admissions from wastewater signal using our modelling approach. In addition, the models built with wastewater only had similar accuracy to models built using clinical data, which means that as clinical data for COVID-19 become less reliable, wastewater is a ready substitute. Wastewater data can be continually collected at low-cost and over large geographies (Galani et al., 2022) and, with lead-time prior to hospital admission (Fernandez-Cassi et al., 2021), models using wastewater for prediction have excellent promise for public health.

The model that we developed retains its relevance in 2023 and beyond despite using data through 2022 because the associations between wastewater detection of SARS-CoV-2 and new hospital admissions remained consistent over time. While the effect size decreased when looking at recent data, the effect remained positive and significant. Changes in the link between detection of virus in wastewater and clinical measures will be subject to many covariates including immunity and variant virulence, meaning that using recent data is going to be essential in applying our modelling method to future prediction of hospital admissions.

Further, we were able to build and test models that can predict specific counts of new hospital admissions at the state, regional, county, and sewershed levels offering health officials a tool they can use to estimate future outcomes. These predictions could inform policy decisions around resource distribution for COVID-19 treatments as well as staff for hospitals prior to and during future surges. Beyond COVID-19, wastewater surveillance has great potential to help in the design and implementation of predictive modelling to inform public health responses to other infectious diseases. Wastewater surveillance can detect most infectious disease pathogens (Kilaru et al., 2023) and our approach for modelling these data could be applied to these other infectious diseases.

Our study focuses on NYS, but the methods used here could be applied to other jurisdictions. Clinical outcomes at the sampling point level might be difficult to obtain, but we still observed good predictive power for models at the state and regional level where clinical data would be more available. Lastly, social vulnerability can be a key factor in negative health outcomes. While this measure was not of high significance in the county level model, at the sewershed level, higher social vulnerability was associated with more hospital admissions. This suggests that social equity is a key issue in COVID-19 hospitalization that should be considered in building predictive models for health outcomes. Public health must consider the social aspects of disease burden to improve outcomes amongst the most vulnerable.

COVID-19 continues to be a leading cause of death and disability in the United States and the world, and accurate prediction of new hospital admissions can improve resource distribution and public health intervention in the face of potential surges. Wastewater concentrations of SARS-CoV-2 gene copies are an accurate predictor of new hospital admissions and provide lead time in NYS up to 10 days in advance. The predictive accuracy of wastewater surveillance is greater than that of clinical surveillance.

## 5 Data sharing statement

Wastewater data used in this analysis are available from the National Wastewater Surveillance System and from the New York State Department of Health at https://health.data.ny.gov/Health/New-York-State-Statewide-COVID-19-Wastewater-Surve/hdxs-icuh. COVID-19 case, hospitalization, and vaccination data are publicly available at the county level from the NYS DOH at https://coronavirus.health.ny.gov/covid-19-data-new-york. Testing data and hospitalization data were obtained through the Electronic Clinical Laboratory Reporting System (ECLRS) (https://www.health.ny.gov/professionals/reportable_diseases/eclrs/) and the Statewide Planning and Research Cooperative System (https://www.health.ny.gov/statistics/sparcs/access/) respectively. Geocoding support and data aggregation at the sewershed level was made possible through the NYS Environmental Public Health tracking program (https://www.health.ny.gov/environmental/public_health_tracking/) System.

R Code used in the analyses is provided in Appendix B.

## CRediT authorship contribution statement

**Dustin T. Hill:** Conceptualization, Data curation, Formal analysis, Investigation, Methodology, Software, Validation, Visualization, Writing – original draft, Writing – review & editing. **Mohammed A. Alazawi:** Conceptualization, Data curation, Writing – review & editing. **E. Joe Moran:** Conceptualization, Data curation, Formal analysis, Methodology, Writing – review & editing. **Lydia J. Bennett:** Data curation, Visualization, Writing – review & editing. **Ian Bradley:** Data curation, Methodology. **Mary B. Collins:** Conceptualization, Funding acquisition, Supervision, Writing – review & editing. **Christopher J. Gobler:** Data curation, Methodology. **Hyatt Green:** Data curation, Funding acquisition, Supervision, Writing – review & editing. **Tabassum Z. Insaf:** Conceptualization, Funding acquisition, Methodology, Project administration. **Brittany Kmush:** Conceptualization, Methodology, Writing – original draft, Writing – review & editing. **Dana Neigel:** Data curation, Visualization, Writing – review & editing. **Shailla Raymond:** Data curation, Visualization, Writing – review & editing. **Mian Wang:** Data curation, Methodology, Writing – review & editing. **Yinyin Ye:** Data curation, Methodology, Writing – review & editing. **David A. Larsen:** Conceptualization, Data curation, Funding acquisition, Methodology, Project administration, Resources, Supervision, Validation, Writing – original draft, Writing – review & editing.

## Declaration of competing interest

The authors declare that they have not competing financial interests.
