# Supplementary material for: Wastewater surveillance provides 10-days forecasting of COVID-19 hospitalizations superior to cases and test positivity: A prediction study
Source: Infect Dis Model. 2023 Oct 31;8(4):1138–50. doi: 10.1016/j.idm.2023.10.004 (PMC10665827; doi:10.1016/j.idm.2023.10.004)
Supplement: Multimedia component 1 [file mmc1.docx]

**Supplemental File 1**

# Supplemental Methods

## Laboratory methods

Wastewater samples were processed and analyzed for SARS-CoV-2 by four regional laboratories each with different methods (see supplemental Table A.1 for brief descriptions). Wastewater methods: processing method and quantification method

| Table A.1: Methods used by regional labs | | |
| --- | --- | --- |
| **Lab** | **Processing method** | **Quantification method** |
| NYC | Centrifuged the PEG precipitation | Reverse transcription quantitative polymerase chain reaction (RT-qPCR) |
| Quadrant | Ultracentrifugation through a sucrose cushion | Reverse transcription quantitative polymerase chain reaction (RT-qPCR) |
| Stony Brook | Centrifuged to remove debris prior to Polyethylene glycol (PEG) precipitation using Quagen QiAamp DSP viral RNA mini kit (Qiagen, Hilden, Germany) | Concentrations measured using digital PCR  NanoDrop One Spectrophotometer (Thermo Fisher Scientific, Waltham, MA.) |
| UB-SUNY 1 | Electronegative membrane filtration | Reverse transcription quantitative polymerase chain reaction (RT-qPCR) |
| UB-SUNY 2 | Nanotrap Magnetic beads | Reverse transcription quantitative polymerase chain reaction (RT-qPCR) |

### NYC

#### Sample processing

Samples in New York City were processed by the NYC lab and consisted of twenty-four hour (24h) flow-weighted composite influent samples. Samples were transported on ice and stored at 4°C before being processed within twelve hours of collection. 40 mL aliquots of each 24h composite sample were pasteurized at 60°C for 90 minutes and then centrifuged to remove solids (5,000 x *g*, 4°C, 10 minutes). Then, the supernatant was filtered using 0.22 µm of cellulose acetate before being subjected to virus concentration using polyethylene glycol (PEG) precipitation. 4 g of PEG and 0.9 g of NaCl were used followed by overnight incubation at 4°C with centrifugation at 12,000 x *g* at 4°C for 120 minutes to pellet the viruses. Once complete, the supernatant was discarded and RNA and DNA were extracted from the concentrated PEG pellet using Qiagen QiAmp Viral RNA Mini Kit with modifications.

#### Quantification method

A real-time quantitative polymerase chain reaction (RT-qPCR) assay was used to quantify copies of the SARS-CoV-2 nucleocapsid or N gene targeting the N1 region in triplicate reactions on a StepOnePlus real-time PCR system from Thermo Fisher Scientific. Please see Hoar et al. 2022 for additional details.^1^

### Quadrant

#### Sample processing

110 mL-1.9L 24h composite samples of influent wastewater were collected then stored at 4°C before being transported to the lab for processing and quantification. Wastewater samples were blended to resuspend particulates that had settled during transport or storage. 20 mL was transferred to an ultracentrifuge tube (Thermo Fisher, MA, USA). A 12 mL sucrose cushion was then added underneath the wastewater using a serological pipette keeping the wastewater and the sucrose solution as distinct layers in the ultracentrifuge tube. These were then ultracentrifuged at 150,000 x *g* at 4°C on a Sorvall® WX Ultra series with a Sorvall SureSpin® 630 Swinginig-Bucekt Rotor for 45 minutes (Thermo Fisher). The resulting pellets with the viral particles and nucleic acids were carefuly decanted with a new pipette and resuspended 200 µL 1X PBS and transferred to 1.7 mL microcentrifuge tubes. These resuspended pellets were stored at -20°C for less than twenty-four hours until nucleic extraction. Extraction was done using the AllPrep® PowerViral® DNA/RNA Kit from Qiagen (Hilden, Germany).

#### Quantification method

RT-qPCR was used to detect the presence of SARS-CoV-2 RNA in undiluted total nucleic extracts using a multiplex reaction with the IP2 and IP4 assays targeting separate regions of the RdRp gene. Thermal cycling for 10 minutes at 50°C, 10 minutes at 95°C, followed by 45 cycles of 95°C for 10 seconds and 59°C for 30 seconds was done. These methods have been previously published and described in detail by Wilder et al. 2022^2^.

### Stony Brook

#### Sample processing

24h composite samples of raw sewage were centrifuged at 4200 rpm for 30 min at 4°C to remove large particles and debris before polyethylene glycol (PEG) precipitation. Recovery rates were evaluated using bovine coronavirus (BCoV), which belongs to the same genus as SARS-CoV-2, was spiked into the supernatant. The viral particles in 40 mL of samples were precipitated with PEG 8000 (Millipore Sigma, Burlington, MA) and NaCl (5 M, Millipore Sigma, Burlington, MA) and then incubated overnight at 4 °C. RNA from the PEG-precipitated wastewater was extracted by Qiagen QIAamp DSP viral RNA mini kit (Qiagen, Hilden, Germany) according to manufacturer’s instructions and eluted in 100 µL by nuclease-free water. The concentrations of RNA were measured by NanoDrop One Spectrophotometer (Thermo Fisher Scientific, Waltham, MA). All RNA samples were stored at −80 °C and subjected to cDNA synthesis within the same day of RNA extraction to avoid losses associated with storing and freezing and thawing RNA extracts.

#### Quantification method

Reverse transcription was performed by High Capacity RNA-to-cDNA Kit (Applied Biosystems, Waltham, MA) at 37 °C for 60 min, and stored at -20 °C until further analysis. The cycling condition was 95 °C for 10 min, followed by 40 cycles of 95 °C for 5 s and 55 °C for 40 s, and 98 °C for 10 min. The total volume of each reaction was 14.5 µL containing 7.25 µL of QuantStudio 3D Digital PCR Master mix v2 (Applied Biosystems, Massachusetts, USA), 0.725 µL of primer and probe (N1/ BCoV), 0.725 µL of TaqMan® Copy Number Reference Assay RNase P (as an internal control, Applied Biosystems, Waltham, MA), 4.8 µL of nuclease-free water, and 1 µL of cDNA template. Digital PCR was performed using N1 primers and probe set from 2019-nCoV CDC EUA Kit (IDT # 10006606) and BCoV set against the BCoV gene as an external reference on a QuantStudio 3D Digital PCR (Applied Biosystems, Massachusetts, USA). Nuclease-free water was used as non-template control (NTC) and plasmids containing the complete nucleocapsid gene from 2019-nCoV (IDT # 10006625) were used as a positive control. Data analysis was performed with the online version of the QuantStudio 3D AnalysisSuite Cloud Software.

### UB-SUNY

#### Sample processing

All samples were 24h composite samples of 100 mL. Samples collected between May 1, 2020 and April 17 2022 were processed using the following method. Method one added magnesium chloride (MgCl_2_) and hydrochloric acid to the sample to adjust the pH to ~3.5. Samples were filtered through 0.45 µm of mixed cellulose esters membranes (Millipore), and then the membranes were collected in cryogenic tubes and saved at -80°C prior to RNA extraction. Extraction occurred using Qiagen PowerMicrobiome Kits on a QIAcube Connect system following the manufacturer’s instructions.

Beginning April 18, 2022, processing method two for UB-SUNY samples took the 24h influent samples of 9.75 mL and mixed them with 100 µL of Nanotrap® Enhancement Reagent 1 (Ceres Nanosciences) and 150 µL of Nanotrap® Microbiome A Particles (Ceres Nanosciences). Viruses were separated from the wastewater using KingFisher Apex Benchtop Sample Prep system from Thermo Fisher. After separation, the nucleic acids were extracted using MagMAX Viral/Pathogen Nucleic Acid Isolation Kits (Thermo Fisher) then eluted in MagMAX Viral/Pathogen Elution Buffer (Thermo Fisher) and stored at -80°C.

#### Quantification method

Samples processed using method one and method two by the UB-SUNY lab were both quantified using the same procedures. UB-SUNY quantified SARS-CoV-2 N gene^3^ using RT-qPCR. The RT-qPCR quantification used 10 µL RT-qPCR reaction mixtures consisting of 5 µL of 2x iTag Universal Probes Reaction Mix from Bio-Rad, 0.25 µL of 50x iScript reverse transcriptase also from Bio-Rad, 0.75 µL of 2019nCoV_N2 (RUO Kit, IDT), and 4µL of undiluted nucleic acid extracts. RT-qPCR of the nucleic acid extracts. The SARS-CoV-2 reactions were heated at 50°C for 15 minutes, 95°C for 1 minute, and 40 cycle of 95°C for 10 seconds and 60°C for 30 seconds. Each RT-qPCR assay was conducted in duplicates or triplicates on a CFX96 Touch Real-Time PCR Detection System (Bio-Rad).

| Table A.2: Descriptive statistics by lab | | | | | |
| --- | --- | --- | --- | --- | --- |
|  | *NYC* | *Quadrant* | *Stony Brook* | *UB-SUNY 1* | *UB-SUNY 2* |
| Sample processing method | PEG precipitation | Ultracentrifugation with sucrose cushion | PEG precipitation | Membrane filtration | Magnetic beads |
| Quantification method | RT-qPCR | RT-qPCR | DD-qPCR | RT-qPCR | RT-qPCR |
| Number of observations | 5,954 | 23,275 | 960 | 4,387 | 962 |
| Number of sewers | 14 | 81 | 4 | 10 | 10 |
| Number of counties | 5 | 46 | 1 | 4 | 4 |
| Number of regions | 1 | 8 | 1 | 1 | 1 |
| Min date | 2020-08-31 | 2020-04-29 | 2020-06-03 | 2020-09-02 | 2022-04-18 |
| Max date | 2022-06-30 | 2022-06-30 | 2022-06-30 | 2022-04-17 | 2022-06-30 |
| Min raw copies/mL | 1.037 | 1.000 | 2.871 | 1.000 | 1.000 |
| Mean raw copies/mL | 11.558 | 67.789 | 111.159 | 18.092 | 448.364 |
| Max raw copies/mL | 144.899 | 3,425.500 | 740.125 | 222.975 | 3,536.753 |
| Min hospital admissions per 100k | 0 | 0 | 0 | 0 | 0 |
| Mean hospital admissions per 100k | 2.043 | 1.416 | 2.094 | 1.936 | 1.445 |
| Max hospital admissions per 100k | 17.378 | 18.899 | 13.906 | 8.706 | 7.818 |

## Clinical data geocoding methods

We cleaned and geocoded the data to points using ESRI ArcGIS, MapMarker, and NYCgbat, with a 97% match rate at street address level. Of 284,772 records, 239,854 street addresses were matched using NYS Street Address Mapping (SAM) locator ^4^ in ESRI ArcGIS with a score of at least 85. All remaining unmatched addresses post- ESRI ArcGIS were run through MapMarker; 32,144 addresses were matched with the result code “S”, which indicates that the record was matched to a single address candidate^5^. The geocoding application offered by NYC department of City Planning, NYgbat,^6^ was used to geocode the remaining unmatched addresses located in NYC; 3,057 addresses were matched.

The remaining 9,717 unmatched addressees (i.e., that were not matched at individual street address level) were further geocoded at zip code level using SAM in ESRI ArcGIS. Out of 9,717 addresses, 9,377 were matched while only 340 addresses were unmatched in which 337 were outside the NYS boundaries.

Before linking the points to sewershed level, we used the spatial parcel data from the NYS Department of Taxation and Finance to exclude the geocoded records covered by private septic (18,704 records). Finally, we linked the dataset to the spatial levels above, excluding those located outside the sewershed or the state boundaries (26,633 records). The exclusion of addresses outside the sewersehd was used for the sewershed level analysis. County, regional, and state models used the total hospital admissions geocoded to those borders both on and off-sewer.

From the Electronic Clinical Laboratory Reporting System (ECLRS) in the NYSDOH, we obtained the COVID-19 testing data for the years 2020- 2022, which includes x, y coordinates. The dataset was processed in R environment to assign the testing results to sewershed, county, regional, and state levels.

## List of holidays and minor holidays used in analysis

We used several major and minor holidays in our model. Holidays were coded and then a week after the holiday was coded to match (a for major, b for minor, and c for not a holiday). These categories reflect time periods for increased human movement and social gathering. Major holidays included: Eid al-Fitr, Eid al-Adha, Rosh Hashanah, Yom Kippur, Hanukkah, Easter, Thanksgiving, and Christmas. Minor holidays included Purim, Passover, New Years Day (January 1), Memorial Day (late May), Independence Day (July 4), and Labor Day (early September).

Also, descriptive statistics for all additional quantitative variables are in Table A.3.

| Table A.3: Descriptive statistics for quantitative variables | | | | | | | |
| --- | --- | --- | --- | --- | --- | --- | --- |
| *Lab* | *Variable* | *n* | *Mean* | *sd* | *Min* | *Median* | *Max* |
| NYC | County asthma rate | 5954 | 11.39 | 1.30 | 10.00 | 12.50 | 12.70 |
|  | County BMI 30^th^ percentile rate | 5954 | 26.19 | 2.56 | 19.90 | 26.90 | 30.10 |
|  | County cancer rate | 5954 | 448.17 | 34.77 | 421.00 | 440.70 | 524.50 |
|  | Proportion of the county with 2 doses of mRNA vaccine | 5954 | 0.49 | 0.26 | 0.00 | 0.58 | 0.85 |
|  | County hospital admissions per 100k population | 5954 | 1.84 | 1.98 | 0.00 | 1.01 | 11.26 |
|  | County respiratory disease rate | 5954 | 24.41 | 3.11 | 20.60 | 26.70 | 27.80 |
|  | SARS-2 raw gene copies | 5954 | 12.50 | 21.59 | 1.00 | 5.60 | 194.98 |
|  | County average social vulnerability | 5954 | 0.57 | 0.11 | 0.32 | 0.56 | 0.80 |
|  | County test positivity | 5954 | 0.06 | 0.10 | 0.00 | 0.03 | 1.00 |
| Quadrant | County asthma rate | 23275 | 5.77 | 4.65 | 2.70 | 4.90 | 33.60 |
|  | County BMI 30^th^ percentile rate | 23275 | 31.71 | 5.81 | 23.80 | 29.60 | 44.80 |
|  | County cancer rate | 23275 | 506.29 | 30.15 | 433.00 | 512.60 | 570.00 |
|  | Proportion of the county with 2 doses of mRNA vaccine | 23275 | 0.25 | 0.28 | 0.00 | 0.05 | 0.75 |
|  | County hospital admissions per 100k population | 23275 | 2.33 | 5.92 | 0.00 | 0.00 | 77.40 |
|  | County respiratory disease rate | 23275 | 24.09 | 6.51 | 14.50 | 24.70 | 58.50 |
|  | SARS-2 raw gene copies | 23275 | 127.70 | 494.30 | 1.00 | 10.60 | 7820.00 |
|  | County average social vulnerability | 23275 | 0.42 | 0.16 | 0.15 | 0.43 | 0.81 |
|  | County test positivity | 23275 | 0.07 | 0.10 | 0.00 | 0.04 | 1.00 |
| Stony Brook | County asthma rate | 446 | 6.60 | 0.00 | 6.60 | 6.60 | 6.60 |
|  | County BMI 30^th^ percentile rate | 446 | 26.10 | 0.00 | 26.10 | 26.10 | 26.10 |
|  | County cancer rate | 446 | 527.40 | 0.00 | 527.40 | 527.40 | 527.40 |
|  | Proportion of the county with 2 doses of mRNA vaccine | 446 | 0.48 | 0.25 | 0.00 | 0.58 | 0.73 |
|  | County hospital admissions per 100k population | 446 | 2.01 | 2.22 | 0.00 | 1.43 | 12.55 |
|  | County respiratory disease rate | 446 | 22.50 | 0.00 | 22.50 | 22.50 | 22.50 |
|  | SARS-2 raw gene copies | 446 | 169.31 | 226.07 | 1.00 | 68.00 | 988.00 |
|  | County average social vulnerability | 446 | 0.29 | 0.00 | 0.29 | 0.29 | 0.29 |
|  | County test positivity | 446 | 0.07 | 0.08 | 0.00 | 0.05 | 0.67 |
| UB-SUNY | County asthma rate | 4141 | 7.50 | 0.00 | 7.50 | 7.50 | 7.50 |
|  | County BMI 30^th^ percentile rate | 4141 | 31.00 | 0.00 | 31.00 | 31.00 | 31.00 |
|  | County cancer rate | 4141 | 539.40 | 0.00 | 539.40 | 539.40 | 539.40 |
|  | Proportion of the county with 2 doses of mRNA vaccine | 4141 | 0.46 | 0.23 | 0.00 | 0.57 | 0.70 |
|  | County hospital admissions per 100k population | 4141 | 1.69 | 2.47 | 0.00 | 0.70 | 20.35 |
|  | County respiratory disease rate | 4141 | 23.00 | 0.00 | 23.00 | 23.00 | 23.00 |
|  | SARS-2 raw gene copies | 4141 | 24.43 | 43.63 | 1.00 | 10.10 | 481.82 |
|  | County average social vulnerability | 4141 | 0.34 | 0.10 | 0.22 | 0.33 | 0.48 |
|  | County test positivity | 4141 | 0.07 | 0.08 | 0.00 | 0.05 | 1.00 |

# Supplemental results

## Trend sensitivity analysis

Trend sensitivity was only slightly influenced by the population served by the treatment plants in the county. Greater population served was moderately correlated with higher sensitivity (Pearson r = 0.33, p = 0.089) and greater PPV (Pearson r = 0.33, p = 0.091) (Table A.4).

| Table A.4: Trend analysis correlations between sensitivity, PPV, specificity, and NPV for each county and the proportion of the population served by wastewater surveillance in that county. | | | |
| --- | --- | --- | --- |
| *Trend threshold* | *Metric* | *Pearson correlation coefficient (r)* | *p value* |
| Greater than or less than 0% | Sensitivity | 0.33 | 0.089• |
|  | PPV | 0.33 | 0.091• |
|  | Specificity | 0.03 | 0.89 |
|  | NPV | 0.10 | 0.61 |
| Greater than or less than 5% | Sensitivity | 0.16 | 0.42 |
|  | PPV | 0.30 | 0.12 |
|  | Specificity | 0.04 | 0.84 |
|  | NPV | -0.04 | 0.84 |
| Greater than or less than 10% | Sensitivity | 0.18 | 0.36 |
|  | PPV | 0.29 | 0.14 |
|  | Specificity | -0.02 | 0.93 |
|  | NPV | -0.07 | 0.72 |
| *Notes: •<0.1* * p value < 0.05, **p < 0.01, *** p < 0.001 | | | |


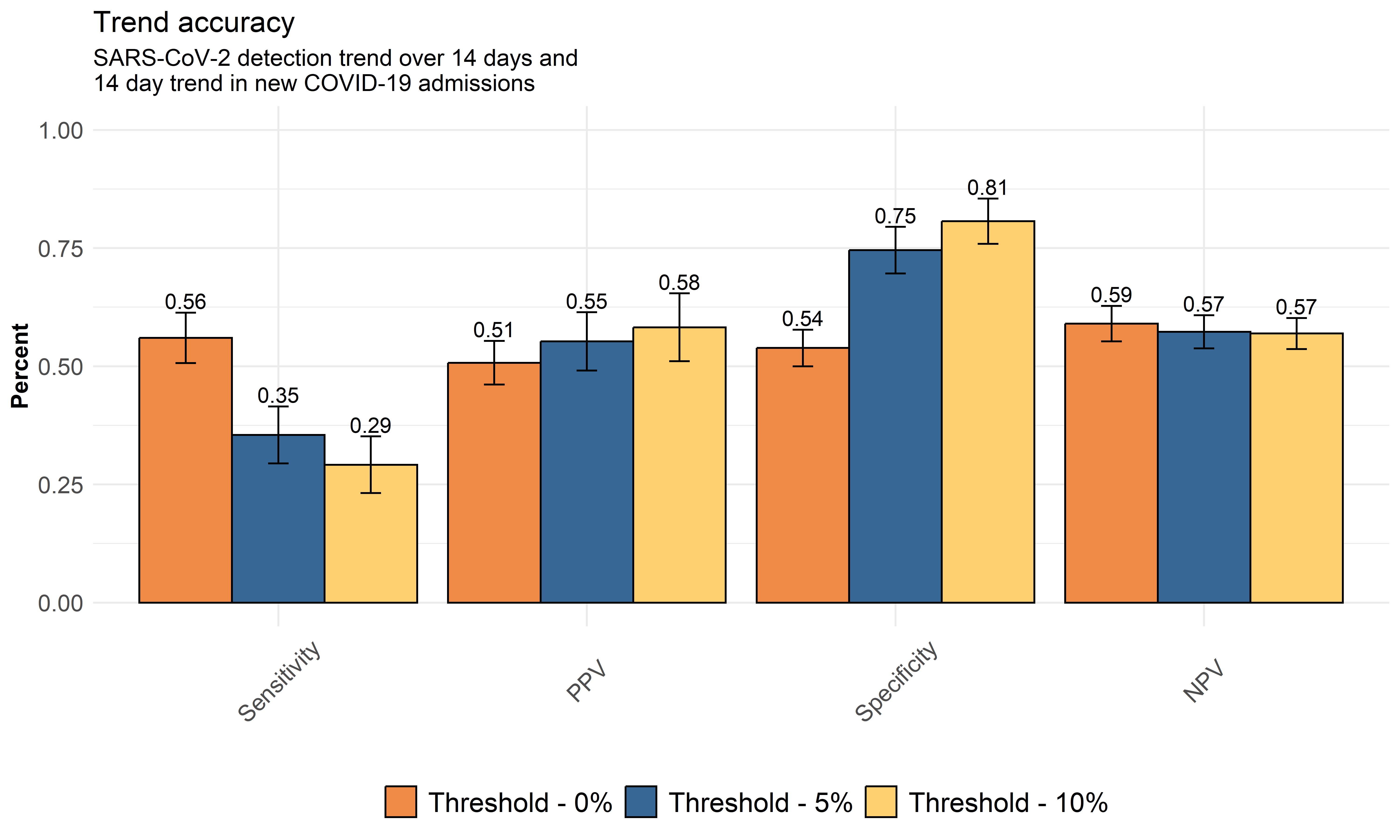


**Figure A1:** Accuracy of the trend in SARS-CoV-2 detection predicting the trend in hospitalizations over 14 days across all counties.

## Correlation between wastewater data and hospital admissions by lab


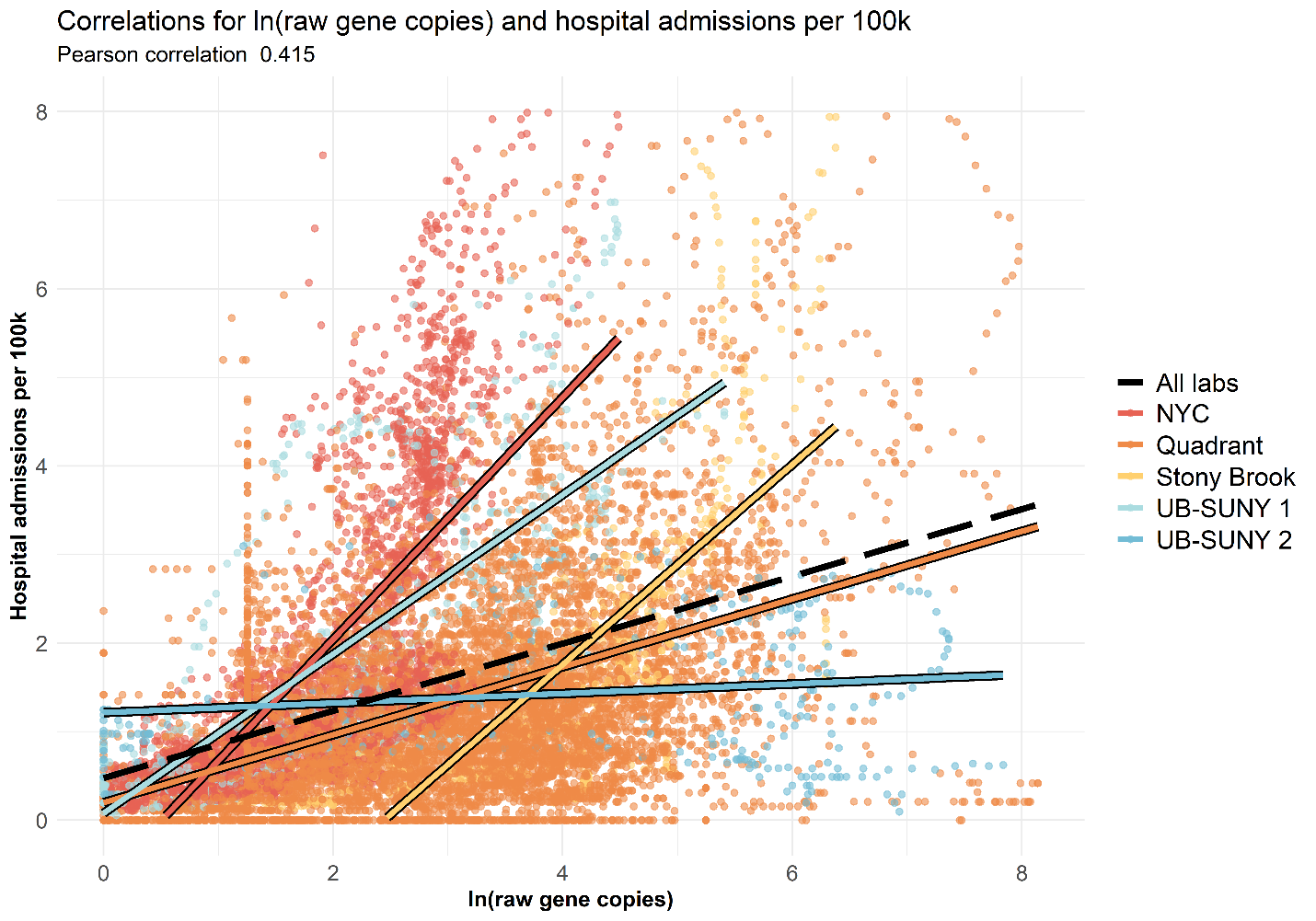


**Figure A2:** Correlations between wastewater the natural log of gene copies and new COVID-19 hospital admissions at the county level.

| Table A.5: Correlation between in-patient hospital admissions per 100k population and ln(SARS-CoV-2 raw gene copies in wastewater) for each lab | | | | | | |
| --- | --- | --- | --- | --- | --- | --- |
|  | All labs | NYC | Quadrant | Stony Brook | UB-SUNY 1 | UB-SUNY 2 |
| Pearson correlation coefficient (r) | 0.415 | 0.72 | 0.44 | 0.69 | 0.73 | 0.19 |
| P value | < 0.001 | <0.001 | <0.001 | <0.001 | <0.001 | <0.001 |

## Statistical Model Evaluation

Of the three aggregation levels, the county proved to the most accurate (MASE 0.39) with the sewershed models being next (MASE 0.56) and the regional models being the least accurate (MASE 1.12). The county model results were the most accurate when including all lab methods and the model was not more accurate when including an auto-regressive component to account for the time series (AR model MASE 0.41). In addition, the model did not need zero-inflation and was not over dispersed. No outliers were detected and all VIF values were below 5. The final, best models for raw copies and intensity were at the county level with a random intercept for county and using a Poisson distribution.


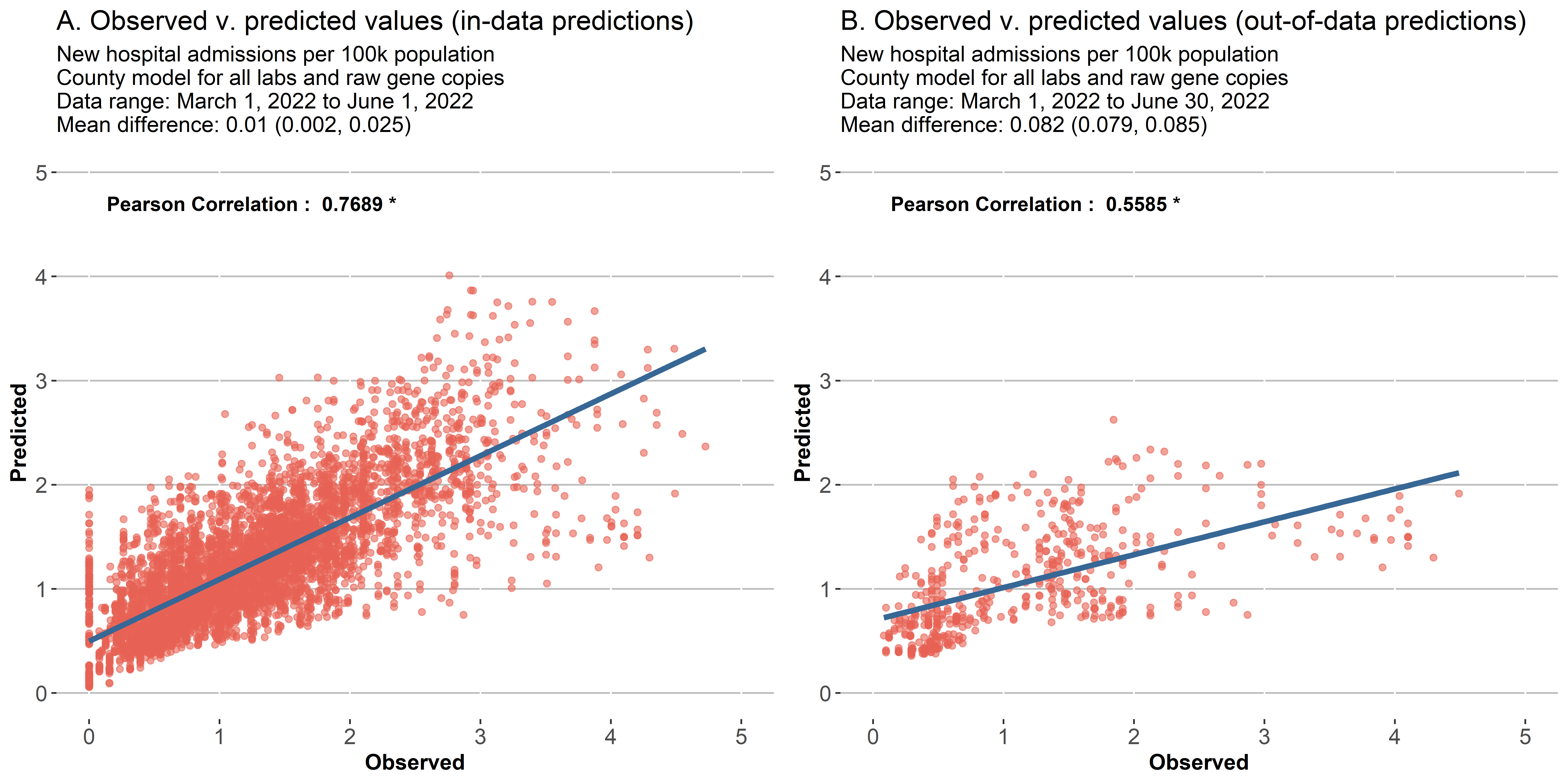


**Figure A.3:** A. Correlation between observed and predicted new COVID-19 hospital admissions per 100,000 the county all labs model for “in data” predictions. B. Correlation between observed and predicted new COVID-19 hospital admissions per 100,000 the county all labs model for “out of data” (new counties and new time period) predictions.

## Spatial and temporal correlation results

Spatial correlation was not detected in the model with a Moran’s I value of -0.0061203 and p value of 0.5302. In addition, temporal correlation was not detected between residuals, and a temporal model with autoregressive covariance was less accurate at model prediction with MASE value of 0.98 versus a model without autoregressive covariance structure with MASE 0.60.

## Results by lab


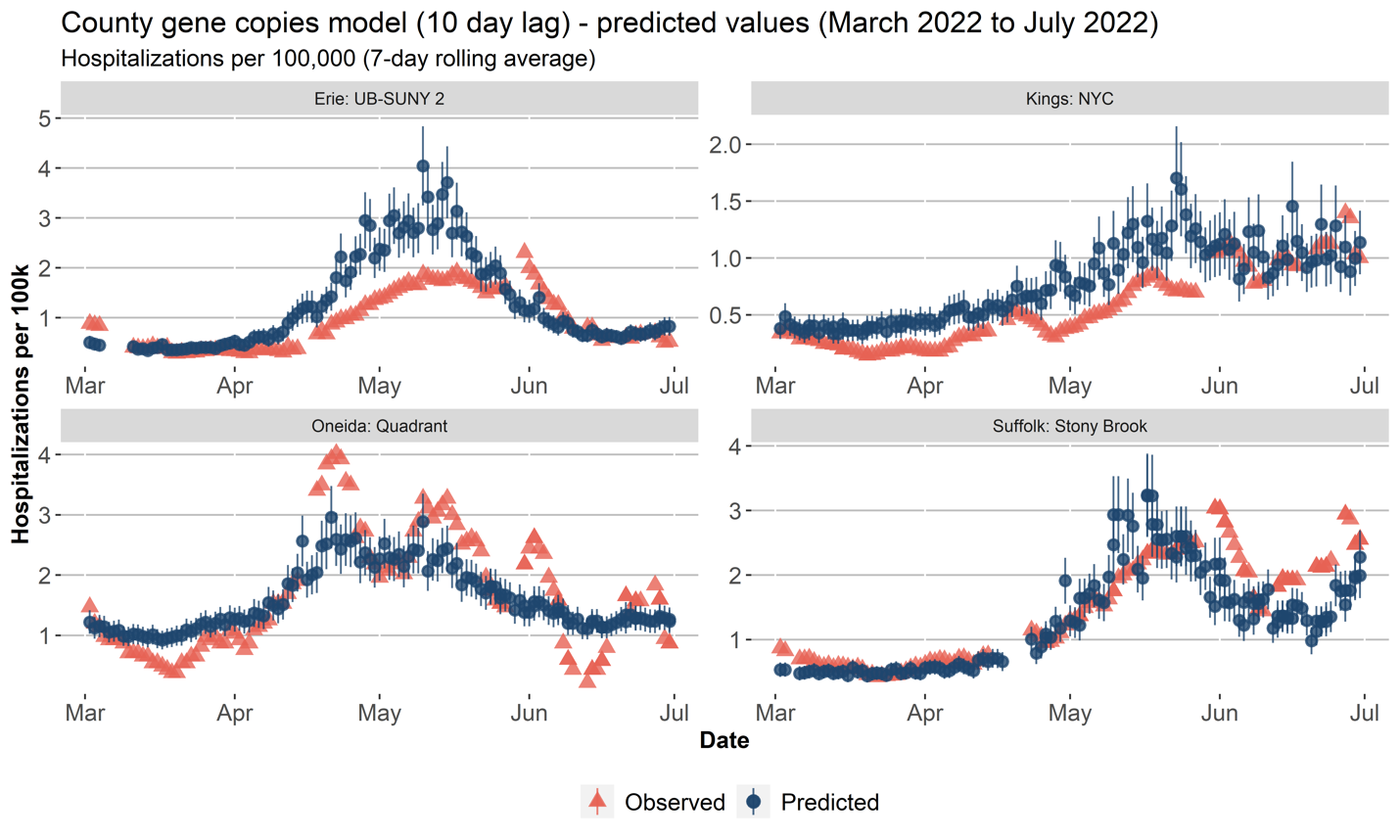


**Figure A.4**: Predicted v. observed new COVID-19 hospital admissions per 100,000 for four select counties with different labs analyzing their results (recent data model).

# Supplemental references

1. Hoar C, Chauvin F, Clare A, et al. Monitoring SARS-CoV-2 in wastewater during New York City’s second wave of COVID-19: sewershed-level trends and relationships to publicly available clinical testing data. *Environ Sci: Water Res Technol*. 2022;8(5):1021-1035. doi:10.1039/D1EW00747E

2. Wilder ML, Middleton F, Larsen DA, et al. Co-quantification of crAssphage increases confidence in wastewater-based epidemiology for SARS-CoV-2 in low prevalence areas. *Water Research X*. 2021;11:100100. doi:10.1016/j.wroa.2021.100100

3. Lu X, Wang L, Sakthivel SK, et al. US CDC Real-Time Reverse Transcription PCR Panel for Detection of Severe Acute Respiratory Syndrome Coronavirus 2. *Emerg Infect Dis*. 2020;26(8):1654-1665. doi:10.3201/eid2608.201246

4. NYS Street Address Mapping (SAM). Accessed January 23, 2023. https://gis.ny.gov/streets/

5. Precisely. Single Match (S category). Accessed January 24, 2023. https://docs.precisely.com/docs/sftw/mapmarker/main/en-us/webhelp/mmo/InputOptions/MMResultCodes_S_category.html

6. Geosupport Desktop Edition^TM^. Accessed January 24, 2023. https://www.nyc.gov/site/planning/data-maps/open-data/dwn-gde-home.page
