# Supplementary material for: Wastewater surveillance provides 10-days forecasting of COVID-19 hospitalizations superior to cases and test positivity: A prediction study
Source: Infect Dis Model. 2023 Oct 31;8(4):1138–50. doi: 10.1016/j.idm.2023.10.004 (PMC10665827; doi:10.1016/j.idm.2023.10.004)

Forecasting New York State COVID-19 Hospitalizations From Wastewater

Dustin T. Hill, Mohammed A. Alazawi, E. Joe Moran, Lydia J. Bennett, Ian Bradley, Mary B. Collins,

2023-06-30

Contents

0.1 Introduction 1
0.2 Data preparation 2
0.2.1 R packages 2
0.2.2 Data prep code 2
0.3 Evaluation of the data and its association with COVID-19 hospital admissions 14
0.3.1 Lag evaluation methods 14
0.3.2 Correlation results across labs 17
0.4 Model fitting 20
0.4.1 Training and testing datasets for each lab 20
0.5 Final model selected 20
0.6 Correlation between predicted and observed 26
0.7 County example plots 32

0.1 Introduction

This document includes the code used to build the current prediction models used for forecasting new COVID-19 in-patient hospital admissions in New York State using wastewater, clinical data, vaccine information, and covariate data.

NOTE The analyses presented here use SPARCS data, however, current hospital forecasting results are based on HERDS data to provide up-to-date results

Further, we use the term forecasting since we are predicting future new hospital admissions in the short-term, but our model is actually built using more traditional predictive modelling approaches rather than time-series forecasting methods. We leverage the lead time between infection and case diagnosis + hospital admission that has been observed with COVID-19 where individuals become infected and start shedding the virus 3-5 days prior to becoming sick and then getting tested. See David A Larsen and others, Coupling freedom from disease principles and early warning from wastewater surveillance to improve health security, PNAS Nexus, Volume 1, Issue 1, March 2022, pgac001, https://doi.org/10.1093/pnasnexus/pgac001 for more information.

0.2 Data preparation

0.2.1 R packages

```
# PACKAGES
library(sf)
library(dplyr)
library(ggplot2)
library(MetBrewer)
library(lubridate)
library(tidyr)

# glmm model packages
library(glmmTMB)
library(bbmle)


# model evaluation
library(performance)
library(yardstick)  # mase with nas
library(hydroGOF)  # mae
library(parameters)  # functions to extract model results, e.g., standard error of betas


# plot helper packages
library(MetBrewer)
library(ggnewscale)

# tables
library(sjPlot)
```

0.2.2 Data prep code

```
# COUNTY DATA ALL COUNTY ADMISSIONS

# county case data
# case data from nys website
all.cases <- read.csv("https://health.data.ny.gov/api/views/xdss-u53e/rows.csv?accessType=DOWNLOAD&bom=
                      header = TRUE, sep = ";")

colnames(all.cases)[1] <- "test_DATE"


# data prep (date variable change and make case data numeric)
all.cases$Date <- mdy(all.cases$test_DATE)
all.cases$Cases <- as.numeric(gsub(",", "", all.cases$New.Positives))


# add test positivity
all.cases$tests <- as.numeric(gsub(",", "", all.cases$Total.Number.of.Tests.Performed))
all.cases$Test_positivity <- all.cases$Cases / all.cases$tests
```

```
# add active cases
all.cases <- all.cases %>%
  group_by(County) %>%
  mutate(active7 = purrr::map_dbl(Date, ~sum(Cases[between(Date, .-7, .)])),
         test.pos.avg7 = purrr::map_dbl(Date, ~mean(Test_positivity[between(Date, .-7, .)], na.rm = TRUE
all.cases <- all.cases %>%
  group_by(County) %>%
  mutate(case.avg7 = purrr::map_dbl(Date, ~mean(Cases[between(Date, .-7, . )], na.rm = TRUE)))

# change st lawrence spelling
all.cases$County[all.cases$County == "St. Lawrence"] <- "St Lawrence"

# fix column names
all.cases$county <- all.cases$County
all.cases$date <- all.cases$Date
all.cases$Tested_Positive <- as.numeric(all.cases$New.Positives)

# select columns we want
cases_county <- all.cases %>%
  ungroup() %>%
  select(date, county, active7, case.avg7, test.pos.avg7, Tested_Positive)
names(cases_county)

# add weekly hopsitalizations by county
assign_week = function(date) {
  weeks = seq.Date(as.Date('2020-01-06'), as.Date('2029-12-31'), by = 7)
  max(weeks[date >= weeks]) |> format('%Y-%m-%d')
}

# add weekly average
cases_week <- cases_county %>%
  group_by(county, week = purrr::map_chr(date, assign_week)) %>%
  summarise(
    w_case_avg = mean(Tested_Positive, na.rm = TRUE)
  )%>%
  ungroup()
cases_week$date <- ymd(cases_week$week)

# merge
cases_county <- left_join(cases_county, cases_week, by = c("date", "county"))
summary(cases_county)

# remove region names from county list
names_remove <- c("Capital Region ", "Central New York", "Long Island", "Mid-Hudson",
                  "Mohawk Valley", "New York City", "North Country",
                  "Southern Tier", "STATEWIDE", "Western New York", "Finger Lakes")
cases_county <- cases_county %>% dplyr::filter(!county %in% names_remove)

#--------------------------------------------------------------------------------
# COUNTY VACCINE DATA

# VACCINATION DATA OVER TIME
vaccine_county <- read.csv("https://health.data.ny.gov/api/views/duk7-xrni/rows.csv?accessType=DOWNLOAD
```

```
                           header = TRUE, sep = ",")

# edit column name
vaccine_county$county <- vaccine_county$County
vaccine_county$county[vaccine_county$county == "St. Lawrence "] <- "St Lawrence"

# change vaccination numbers to percentages of county population
# load county population estimates
crosswalk <- read.csv("Data/State_files/Crosswalks/NY_Sewer_To_County_Crosswalk.csv", stringsAsFactors =
#County level multiplier for per 100k clculations
pop <- crosswalk
pop$county.prop<-100000/pop$County_pop
# change column name
pop$county <- pop$Home_County
# select columns we want to keep
pop <- pop %>% select(county, County_pop, county.prop) %>% distinct()

# merge
vaccine_county <- left_join(vaccine_county, pop, by = c("county"))

# calculate vaccination percentages
vaccine_county$Partially_vaccinated_county_prop <- vaccine_county$First.Dose / vaccine_county$County_pop
vaccine_county$Fully_vaccinated_county_prop <- vaccine_county$Series.Complete / vaccine_county$County_po
summary(vaccine_county)
# make date column
vaccine_county$date <- mdy(vaccine_county$Report.as.of)

# select data we want
vaccine_county <- vaccine_county %>% select(Region, county, date, County_pop, Partially_vaccinated_count
                                            Fully_vaccinated_county_prop)
summary(vaccine_county)

#-------------------------------------------------------------------------------
# COUNTY HOSPITALIZATIONS - > geocode only

hospital1 <- read.csv("Data/Hospitalizations/HOSPT_COUNTY_DATE_FINAL_ALL_2020_2021_I_Dec25_22.csv",
                      stringsAsFactors = FALSE,
                     header=T, sep=",", na.strings=c("","NA"))

hospital2 <-read.csv("Data/Hospitalizations/HOSPT_COUNTY_DATE_FINAL_ALL_JAN_DEC_2022_I.csv",
                     stringsAsFactors = FALSE,
                     header=T, sep=",", na.strings=c("","NA"))
hospital <- bind_rows(hospital1, hospital2)

names(hospital)
head(hospital)

# edit column names and details
hospital$date <- ymd(hospital$ADMIT_DT)
hospital$county <- hospital$COUNTY
hospital$hos.count <- hospital$COUNT
summary(hospital)
```

```r
# fix st lawrence
hospital$county[hospital$county == "St. Lawrence"] <- "St Lawrence"

# fill in missin dates
hospital<- hospital %>%
  dplyr::group_by(county) %>%
  complete(date = seq.Date(min(date), max(date), by = "day")) %>%
  mutate(max_date = max(date)) %>%
  ungroup()

# replace missing values with 0
hospital$hos.count <- ifelse(is.na(hospital$hos.count), 0, hospital$hos.count)

#moving averages for hospitalizations - 7 day avg is useful if we only sample 1x/week
hospital<- hospital %>%
  dplyr::arrange(date)%>%
  dplyr::group_by(county) %>%
  dplyr::mutate(hos.7avg = purrr::map_dbl(date, ~mean(hos.count[between(date, .-7, .)], na.rm = TRUE)))%
  dplyr::ungroup()
summary(hospital)

# select columns
hospital <- hospital %>% select(date, county, hos.count, hos.7avg, max_date)

hospital_week <- hospital %>%
  group_by(county, week = purrr::map_chr(hospital$date, assign_week)) %>%
  summarise(
    w_hosp_avg = mean(hos.count, na.rm = TRUE)
  )%>%
  ungroup()
hospital_week$date <- ymd(hospital_week$week)
hospital <- left_join(hospital, hospital_week, by = c("date", "county"))
summary(hospital)

# data up to june 30th
hospital <- hospital %>%
  dplyr::filter(date <= "2022-06-30")

#-------------------------------------------------------------------------------

# WASTEWATER DATA - COUNTY AGGREGATION
# data
ww.data <- aws.s3::s3readRDS(object = 'nys.wastewater.rds',
                               bucket = 'nystatewws',
                              key = Sys.getenv('AWS_KEY'),
                            secret = Sys.getenv('AWS_SECRET'))

ww.data$date<-ww.data$sample_collect_date

# drop pilot data
ww.erie <- ww.data %>%
  dplyr::filter(county == "Erie")
ww.erie <- ww.erie %>% dplyr::filter(!is.na(lab_id))
```

```
ww.data <- ww.data %>% dplyr::filter(!county == "Erie")
ww.data <- rbind(ww.data, ww.erie)

ww.data$county[ww.data$county == "St. Lawrence"] <- "St Lawrence"

# remove weird date
ww.data <- ww.data %>% dplyr::filter(date > "2020-01-01")

# remove upstream sampling points
ny_sewersheds <- st_read("E:/Dropbox/CEMI/Wastewater/Data/State_files/New York State Sewersheds/New York
upstream <- ny_sewersheds %>% dplyr::filter(!Method == "Influent" | is.na(Method))

# remove binghampton from the upstream list
upstream <- upstream %>% dplyr::filter(!WWTP_ID == "NY0024414")

# remove upstream
ww.data <- ww.data %>% dplyr::filter(!sw_id %in% upstream$SW_ID)

# add suffolk  bergen point data
suffolk <- read.csv("E:/Dropbox/CEMI/Wastewater/Data/Master Database/Suffolk_SD3_summary 2020-2022.csv"
                    stringsAsFactors = FALSE)
# edit column names
suffolk$date <- mdy(suffolk$Date)
suffolk$pcr_target_avg_conc <- as.numeric(suffolk$N1.gene.copy.per.liter)/1000
suffolk$sw_id <- "36103NY0104809SCWWA"
suffolk$lab_id <- "stonybrook"
suffolk$county <- "Suffolk"
ww.data <- bind_rows(ww.data, suffolk)

suffolk_test <- ww.data %>% dplyr::filter(county == "Suffolk")
summary(suffolk_test$date)

ww.data<-ww.data%>% dplyr::select(date, sw_id, county, facility,sars_pos, pcr_target_avg_conc, hum_frac
                                  flow_rate, lab_id)

# Calculating copies of SARS2 RNA #
ww.data$sars2 <- 3.5 # 5/sqrt(2)
ww.data$sars2 <- ifelse(!is.na(ww.data$pcr_target_avg_conc) & ww.data$pcr_target_avg_conc > 0, as.numer
ww.data$sars2 <- ifelse(ww.data$sars_pos==0, 1, ww.data$sars2)
ww.data$sars2 <- ifelse(is.na(ww.data$sars2), as.numeric(as.character(ww.data$pcr_target_avg_conc)),
                        ww.data$sars2)
ww.data$sars2 <- ifelse(!is.na(ww.data$pcr_target_avg_conc) & ww.data$pcr_target_avg_conc == 0, 1,
                        ww.data$sars2)
summary(ww.data$sars2)

# check western data loq
min(ww.data$sars2, na.rm = TRUE) #

# some nas still, coming from NYC data
t <- ww.data %>% dplyr::filter(is.na(sars2))
# drop those data points because we do not know of they are true 0s or not
ww.data <- ww.data %>% dplyr::filter(!is.na(sars2))
ww.data$sars2[ww.data$pcr_target_avg_conc == 0] <- 1
```

```
ww.data$sars2 <- ifelse(ww.data$sars2 == 0, 1, ww.data$sars2)
# less than 1 assign 0
ww.data$sars2[ww.data$sars2 < 1] <- 1
summary(ww.data$sars2)

# calculate intensity
ww.data = ww.data |>
  mutate(
    intensity = case_when(
      sars_pos == 0 ~ 0, # True zeros are 0 intensity (includes Buffalo)
      is.na(hum_frac_mic_conc) ~ NA_real_, # Any samples missing hum_frac_mic_conc set to NA for now
      lab_id == 'UB-SUNY' & is.na(pcr_target_avg_conc) ~ log(4/sqrt(2)), # Buffalo <LOQ
      lab_id == 'NYC' & is.na(pcr_target_avg_conc) ~ 0, # NYC <LOD & <LOQ are both 0
      sars_pos < 3 | (is.na(sars_pos) & is.na(pcr_target_avg_conc)) | is.na(pcr_target_avg_conc) ~ log([illegible]
      TRUE ~ log(pcr_target_avg_conc)/log(hum_frac_mic_conc) # Otherwise standard calculation
    )
  )
summary(ww.data$intensity)

#SEWERSHED POPULATION DATA
ww.stats <- read.csv("Data/State_files/NY_Sewershed-Population_And_Treatment_Plant_Stats.csv",
                     stringsAsFactors = FALSE, header=T, sep=",", na.strings=c("","NA"))

#remove upstream so we don't double count
ww.stats<-subset(ww.stats, sample_location=="wwtp")
ww.stats<-ww.stats%>% dplyr::select(county_names, SW_ID, population_served)
names(ww.stats)<-c("county", "sw_id", "pop.served")

#sewershed pop proportion for 100k normalization multiplier
ww.stats$sw.prop<-100000/ww.stats$pop.served

ww.data <- left_join(ww.data, ww.stats, by = c("sw_id", "county"))

# remove labs and b
labs_remove <- c("A", "B")
ww.data <- ww.data %>% dplyr::filter(!lab_id %in% labs_remove)
summary(ww.data$intensity)

# COUNTY AGGREGATION OPTION 1 : 7 DAY AVERAGE PER SITE THEN WEIGHT TO COUNTY LEVEL BY DAY

# fill in missing dates
ww.county <- ww.data %>%
  dplyr::group_by(sw_id, county, pop.served) %>%
  complete(date = seq.Date(min(date), max(date), by = "day")) %>%
  ungroup()

# linear approximation for wastewater data
ww.county <- ww.county %>%
  group_by(sw_id, county, pop.served) %>%
  dplyr::arrange(date)%>%
  dplyr::mutate(intensity.approx_swid = na.approx(intensity, maxgap = 8, na.rm = FALSE),
                sars2.approx_swid = na.approx(sars2, maxgap = 8, na.rm = FALSE)
  )%>%
```

```
  dplyr::ungroup()

# rolling average per site
ww.county <- ww.county %>%
  group_by(sw_id, county, pop.served) %>%
  dplyr::arrange(date)%>%
  dplyr::mutate(intensity.7avg_swid = purrr::map_dbl(date, ~mean(intensity[between(date, .-7, .)],
                                                                     na.rm = TRUE)),
                sars2.7avg_swid = purrr::map_dbl(date, ~mean(sars2[between(date, .-7, .)],
                                                                 na.rm = TRUE)),
                intensity.approx.7avg_swid = purrr::map_dbl(date, ~mean(intensity.approx_swid[between(da
                                                                            na.rm = TRUE)),
                sars2.approx.7avg_swid = purrr::map_dbl(date, ~mean(sars2.approx_swid[between(date, .-7
                                                                        na.rm = TRUE))
                )%>%
  dplyr::ungroup()
summary(ww.county)

# aggregate to county with weighted mean
ww.county <- ww.county %>%
  dplyr::filter(!is.na(sw_id))%>%
  group_by(date, county)%>%
  mutate(intensity.7avg = weighted.mean(intensity.7avg_swid, pop.served, na.rm = TRUE),
         sars2.7avg = weighted.mean(sars2.7avg_swid, pop.served, na.rm = TRUE),
         intensity.approx.7avg = weighted.mean(intensity.approx.7avg_swid, pop.served, na.rm = TRUE),
         sars2.approx.7avg = weighted.mean(sars2.approx.7avg_swid, pop.served, na.rm = TRUE)
  )%>%
  ungroup()

# select what we want to keep and remove duplicate rows
ww.county <- ww.county %>% select(date, county, lab_id, intensity.approx.7avg, sars2.approx.7avg,intens
                                  sars2.7avg) %>% distinct()
summary(ww.county)

# rename data -> will compare each one to see which is the better for prediction
colnames(ww.county) <- c("date", "county", "lab_id", "intensity.approx.7avg", "sars2.approx.7avg",
                         "intensity.7avg_orig", "sars2.7avg_orig")

ww.data$week = purrr::map_chr(ww.data$date, assign_week)

# assuming intensity is already calculated
ww.week <-
  ww.data |>
  dplyr::filter(!is.na(intensity), !is.na(sw_id)) |>
  dplyr::filter(between(intensity, 0, 1)) |>  # still some oddities
  left_join(ny_sewersheds |> select(sw_id = SW_ID, population = POP2020)) |>
  dplyr::filter(!is.na(population)) |>  # older samples with no match

  group_by(week, sw_id) |>
  mutate(intensity = mean(intensity),
         sars2 = mean(sars2)) |> # account for multiple samples per sw_id per week
  ungroup() |>
  distinct(week, county, population, intensity, sars2) |>  # reduce to single record per sw_id per week
```

```
  group_by(week, county) |>
  summarise(intensity_week = weighted.mean(intensity, population),
            sars2_week = weighted.mean(sars2, population)) |>
  ungroup()

ww.week$date <- ymd(ww.week$week)

# merge to the main dataset
ww.county <- left_join(ww.county, ww.week, by = c("county", "date"))
summary(ww.county)

#--------------------------------------------------------------------------------
# COUNTY COVARIATES AND COMORBIDITIES

#COMORBITITIES
comorbid<-read.csv("Data/Comorbidities/Risk Factor Long Dataset - County.csv", header=T, sep=",",
                   na.strings=c("","NA"))
comorbid$County<-removeWords(comorbid$County, " County")
colnames(comorbid)[1]<-"county"
comorbid$svi_county <- comorbid$RPL_THEMES
comorbid$county[comorbid$county == "St. Lawrence"] <- "St Lawrence"

# select the key fields we want for now. Can return to this if we need more covariates
comorbid <- comorbid %>% dplyr::select(county, CancerRate, AsthmaRate, CDKRateA, RespRate, BMI25Rate,
                                       BMI30Rate, svi_county)

#--------------------------------------------------------------------------------

# COUNTY CENSUS DEMOGRAPHICS

county_census <- read.csv("E:/Dropbox/CEMI/Wastewater/Data/State_files/county_census.csv",
                          stringsAsFactors = FALSE)

colnames(county_census)[1] <- "county"


#--------------------------------------------------------------------------------
#CREATE HOLIDAY DATA
#do we want a categorical variable of not holiday / holiday  - or -
#do we want numerical factors na (not holiday), 0 (week prior to holiday), 1(week of holiday), 2(week a

#List of holiday dates:
#smaller holidays - new years eve, memorial day, 4th of july, labor day,
#family gathering holidays - easter, thanksgiving, christmas
#other ethnic holidays?

major20<-ymd(c("2020-04-12", "2020-11-26", "2020-12-25",
               "2020-07-30", "2020-05-23",
               "2020-09-18", "2020-12-14", "2020-09-27"))
major21<-ymd(c("2021-04-04", "2021-11-25", "2021-12-25",
               "2021-05-12", "2021-07-19",
               "2021-09-06", "2021-12-02", "2021-09-15"))
minor20<-ymd(c("2020-12-31", "2020-05-25", "2020-07-04", "2020-09-07",
```

```r
                "2020-03-09", "2020-04-08"))
minor21<-ymd(c("2021-12-31", "2021-05-31", "2021-07-04","2021-09-06",
                "2021-02-25", "2021-03-27"))

major22<-ymd(c("2022-04-17", "2022-11-24", "2022-12-25",
                "2022-05-01", "2022-07-08",
                "2022-09-25", "2022-12-22", "2022-10-04"))
minor22 <-ymd(c("2022-12-31", "2022-05-30", "2022-07-04","2022-09-05",
                 "2022-03-16", "2022-04-05"))

major<-as.data.frame(c(major20,major21,major22))
colnames(major)[1]<-"date"
major$category<-"a"
minor<-as.data.frame(c(minor20,minor21, minor22))
colnames(minor)[1]<-"date"
minor$category<-"b"
holidays<-as.data.frame(rbind(major,minor))
holidays$date<-as.Date(holidays$date)
holidays$upper<-holidays$date+7
holidays$id <- seq_along(holidays[,1])

#make week range associated with holidays
holidays<-holidays %>%
  group_by(id)%>%
  mutate(new.dates = list(seq(date, upper, by='day')))%>%
  unnest() %>%
  ungroup()
holidays$date<-holidays$new.dates
holidays<-holidays%>%dplyr::select(id, category, date)
summary(holidays)

#--------------------------------------------------------------------------------
# MERGE TOGETHER
#Sewershed level merges (cases, ww, metadata, hospitalization, vaccination)
# merge one at a time
data.county <- left_join(cases_county, vaccine_county, by = c("county", "date"))
data.county <- left_join(data.county, hospital, by = c("county", "date"))
data.county <- left_join(data.county, ww.county, by = c("county", "date"))
data.county <- left_join(data.county, comorbid, by = c("county"))

# add holidays
data.county <- left_join(data.county, holidays, by = c("date"))

# add covariates from census
data.county <- left_join(data.county, county_census, by = c("county"))

# trim data to stop at max date for each county
data.county <- data.county %>%
  group_by(county) %>%
  dplyr::filter(date <= max_date) %>%
  ungroup()

summary(data.county)
```

```
# check date range
summary(data.county$date)

# nas in comorbidities?
na <- data.county %>% dplyr::filter(is.na(RespRate))
table(na$county)

# add days with no holidy as category c

data.county$category <- ifelse(is.na(data.county$category), "c", data.county$category)
table(data.county$category)

# missing county population for a bunch of records
county_pop_df <- data.county %>%
  group_by(county) %>%
  summarise(county_pop2 = mean(County_pop, na.rm = TRUE)
  ) %>%
  ungroup()
summary(county_pop_df)
# add st lawrence pop 108352
county_pop_df$county_pop2[county_pop_df$county == "St Lawrence"] <- 108352
# merge to data and replace missing values with these ones
data.county <- left_join(data.county, county_pop_df, by = c("county"))
data.county$County_pop <- ifelse(is.na(data.county$County_pop), data.county$county_pop2, data.county$Cou[illegible]
summary(data.county$county_pop2)
na <- data.county %>% dplyr::filter(is.na(County_pop))
data.county$County_pop <- ifelse(is.na(data.county$County_pop), 108352, data.county$County_pop)
table(na$county)
summary(data.county$County_pop)

# add county.prop
data.county$county.prop <- 100000/data.county$County_pop
summary(data.county$county.prop)

# fix missing regions
na_region <- data.county %>% dplyr::filter(is.na(Region))
table(na_region$county)

#counties by region
regions<-read.csv("Data/County_Region.csv", header=T, sep=",", na.strings=c("","NA"))
# select columns to keep
regions <- regions %>% select(region, county)
# fix st lawrence
regions$county[regions$county == "St. Lawrence"] <- "St Lawrence"

# join
data.county <- left_join(data.county, regions, by = c("county"))
summary(data.county$region)
na <- data.county %>% dplyr::filter(is.na(region))

# drop extra region column
data.county <- data.county %>% select(-Region)
table(data.county$region)
```

```
# na vaccinations should be 0
data.county$Partially_vaccinated_county_prop <- ifelse(is.na(data.county$Partially_vaccinated_county_pro
data.county$Fully_vaccinated_county_prop <- ifelse(is.na(data.county$Fully_vaccinated_county_prop), 0,
                                                    data.county$Fully_vaccinated_county_prop)

# check lb ids
table(data.county$lab_id)

# fill missing lab id
ub <- c("Erie", "Chautauqua", "Cattaraugus", "Allegany", "Niagara")
nyc <- c("New York", "Richmond", "Bronx", "Kings", "Queens")
Stonybrook <- c("Suffolk")
gohealth <- c("Orleans", "Genesee")
other_labs <- c("Erie", "Chautauqua", "Cattaraugus", "Allegany", "Niagara", "New York", "Richmond",
                "Bronx", "Kings", "Queens", "Suffolk",
                "Orleans", "Genesee")

data.county$lab_id_r <- ""
data.county$lab_id_r <- ifelse(data.county$county %in% ub , "UB-SUNY", data.county$lab_id_r)
data.county$lab_id_r <- ifelse(data.county$county %in% nyc , "NYC", data.county$lab_id_r)
data.county$lab_id_r <- ifelse(data.county$county %in% Stonybrook , "Stony Brook", data.county$lab_id_r
data.county$lab_id_r <- ifelse(data.county$county %in% gohealth , "GO-Health", data.county$lab_id_r)
data.county$lab_id_r <- ifelse(!data.county$county %in% other_labs , "Quadrant", data.county$lab_id_r)

# ub suny 1 and 2
data.county$lab_id_r <- ifelse(data.county$lab_id_r == "UB-SUNY" & data.county$date <= "2022-04-17", "U
                               data.county$lab_id_r)
data.county$lab_id_r <- ifelse(data.county$lab_id_r == "UB-SUNY" & data.county$date > "2022-04-17", "UB
                               data.county$lab_id_r)

table(data.county$lab_id_r)
table(data.county$lab_id)

# add county prop over time
county_prop_time <- read.csv("Papers/Modelling hospitalizations/Data/county_prop_time.csv")
county_prop_time$date <- ymd(county_prop_time$date)
data.county <- left_join(data.county, county_prop_time, by = c("county", "date"))

# add season covariates
winter <- c(11, 12, 1, 2, 3)
summer <- c(4,5,6,7,8,9,10)
data.county$season_2 <- ifelse(month(data.county$date) %in% winter, "Winter", "Summer")
table(data.county$season_2)

winter <- c(12,1,2)
spring <- c(3, 4,5)
summer <- c(6,7,8)
fall <- c(9, 10, 11)
data.county$season_4 <- ""
data.county$season_4[month(data.county$date) %in% winter] <- "Winter"
data.county$season_4[month(data.county$date) %in% spring] <- "Spring"
data.county$season_4[month(data.county$date) %in% summer] <- "Summer"
data.county$season_4[month(data.county$date) %in% fall] <- "Fall"
```

```r
table(data.county$season_4)

# save county data
saveRDS(data.county, "Papers/Modelling hospitalizations/Data/county_data_hosp_county_full.rds")
```

0.2.2.1 Data included in the analysis The following counties and treatment plants are included in the final analysis. The final model fitting used data from April 2020 to June 2022.

The peak is close to 10 days particularly for log(raw copies), which is what we use for most of the modelling since many labs and counties lack fecal indicator data for normalization.

```
## Reading layer ‘New York State sewersheds’ from data source
##   ‘E:\Dropbox\CEMI\Wastewater\Data\State_files\New York State Sewersheds\New York State sewersheds.s
##   using driver ‘ESRI Shapefile’
## Simple feature collection with 675 features and 25 fields
## Geometry type: MULTIPOLYGON
## Dimension:     XY
## Bounding box:  xmin: -79.72533 ymin: 40.49584 xmax: -72.29054 ymax: 45.01053
## Geodetic CRS:  NAD83

## Reading layer ‘Counties_Shoreline’ from data source
##   ‘E:\Dropbox\CEMI\Wastewater\Data\NYS_Civil_Boundaries.shp’
##   using driver ‘ESRI Shapefile’
## Simple feature collection with 62 features and 17 fields
## Geometry type: MULTIPOLYGON
## Dimension:     XY
## Bounding box:  xmin: 105571.4 ymin: 4483090 xmax: 764142.8 ymax: 4985444
## Projected CRS: NAD83 / UTM zone 18N
```

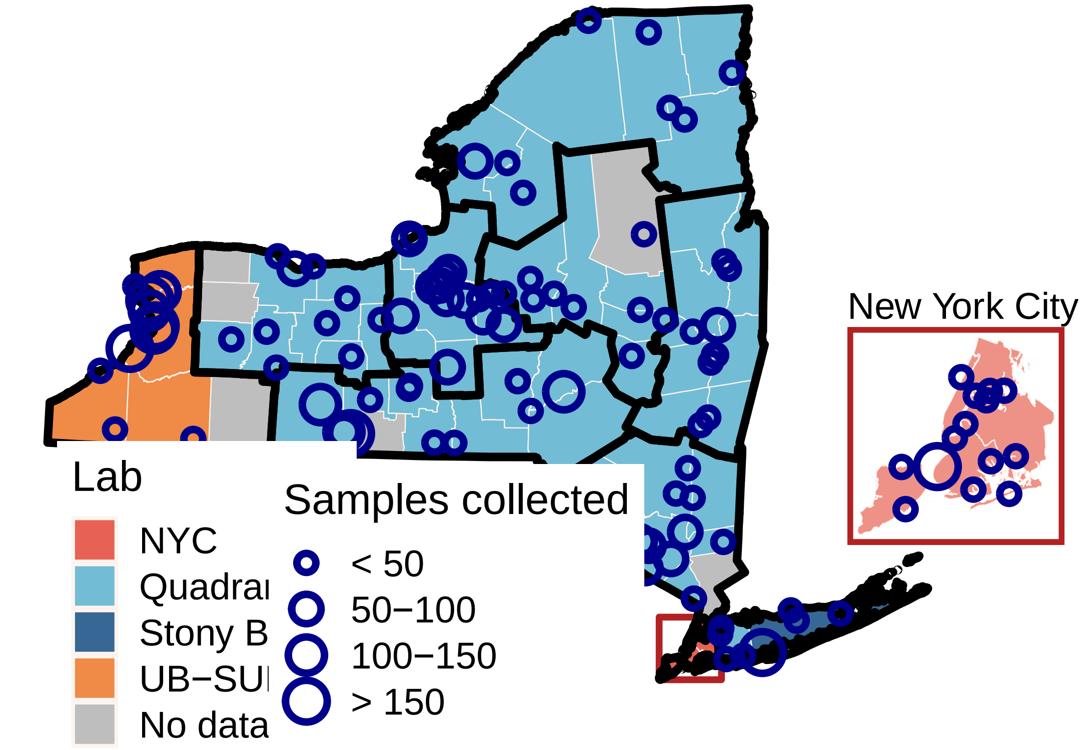

White lines are county borders, thick black lines are NY Economic Development Council Regions.

0.3 Evaluation of the data and its association with COVID-19 hospital admissions

0.3.1 Lag evaluation methods

Finding the optimal lead time for wastewater quantification results and new hospital admissions using Pearson correlation values.

```
# all hospitals
data.county <- readRDS("E:/Dropbox/CEMI/Wastewater/Papers/Modelling hospitalizations/Data/county_data_h

# FIND THE BEST LAGS FOR WASTEWATER AND CASES
### GLOBAL CORRELATIONS FOR BEST LAG FOR ALL DATA ###

# create one lag for daily data and test correlation
# empty list
datalist_lags <- list()

# number of lags to test
c <- 1:30

# for loop for daily lags
for(i in c){
  # create one lag for daily data and test correlation
  data.county <- data.county %>%
```

```
  group_by(county) %>%
  arrange(-desc(date)) %>%
  dplyr::mutate(
    intensity.7avg_lag = lag(intensity.approx.7avg, i, default = NA),
    sars2.7avg_lag = lag(sars2.approx.7avg, i, default = NA),
    cases.avg7_lag = lag(case.avg7, i, default = NA),
    test.pos.avg7_lag = lag(test.pos.avg7, i, default = NA)

  )%>%
  ungroup()

# 7 day average
# store lag
lag_n <- i
# correlation
cor_result_7avg <- cor(data.county$intensity.7avg_lag, data.county$hos.7avg*data.county$county.prop, [illegible]

group2 <- "Intensity Avg 7"

# dataframe of values
df_7avg<- as.data.frame(cbind(group2, lag_n, cor_result_7avg))
colnames(df_7avg) <- c("group", "lag_n", "cor_result")

# gene copies - avg

# correlation
cor_result_sars2 <- cor(log(data.county$sars2.7avg_lag), data.county$hos.7avg*data.county$county.prop
                        use = "na.or.complete")

group4 <- "log(copies Avg 7)"

# dataframe of values
df_7sars<- as.data.frame(cbind(group4, lag_n, cor_result_sars2))
colnames(df_7sars) <- c("group", "lag_n", "cor_result")


# correlation
cor_result_7avg <- cor(data.county$cases.avg7_lag, data.county$hos.7avg*data.county$county.prop,
                       use = "na.or.complete")

group7 <- "Cases Avg 7"

# dataframe of values
df_7avg_case<- as.data.frame(cbind(group7, lag_n, cor_result_7avg))
colnames(df_7avg_case) <- c("group", "lag_n", "cor_result")

# test pos
cor_result_7avg <- cor(data.county$test.pos.avg7_lag, data.county$hos.7avg*data.county$county.prop,
                       use = "na.or.complete")

group7 <- "Test positivity Avg 7"

# dataframe of values
```

```r
  df_7avg_tp<- as.data.frame(cbind(group7, lag_n, cor_result_7avg))
  colnames(df_7avg_tp) <- c("group", "lag_n", "cor_result")

  # combine all metrics
  df <- rbind(df_7avg,  df_7sars,  df_7avg_case, df_7avg_tp)

  datalist_lags[[i]] <- df
}
daily_global_lags <- do.call(rbind, datalist_lags)


# plot them
pal <- met.brewer(name = "Hiroshige", n = 8)

group_keep <- c("")
#daily_global_lags <- daily_global_lags %>% filter(group %in% group_keep)

correlations <-
  ggplot(data = daily_global_lags, aes(x = as.numeric(lag_n), y = as.numeric(cor_result), color = group
                                       fill = group))+
  geom_point(size = 2.5)+
  geom_line(size = 1)+
  theme_bw()+
  theme(
    axis.text=element_text(size=12),
    axis.title=element_text(size=12,face="bold"),
    plot.title = element_text(size=15),
    plot.subtitle = element_text(size = 12),
    plot.caption = element_text(size = 11),
    legend.text = element_text(size = 14),
    legend.title = element_blank())+
  labs(x = "Lag",
       y = "Correlation",
       title = "Pearson correlation values",
       subtitle = "Correlation with hospital admission per 100,000 (all county COVID admission)"
  )+
  scale_color_manual(values = pal)
correlations
```

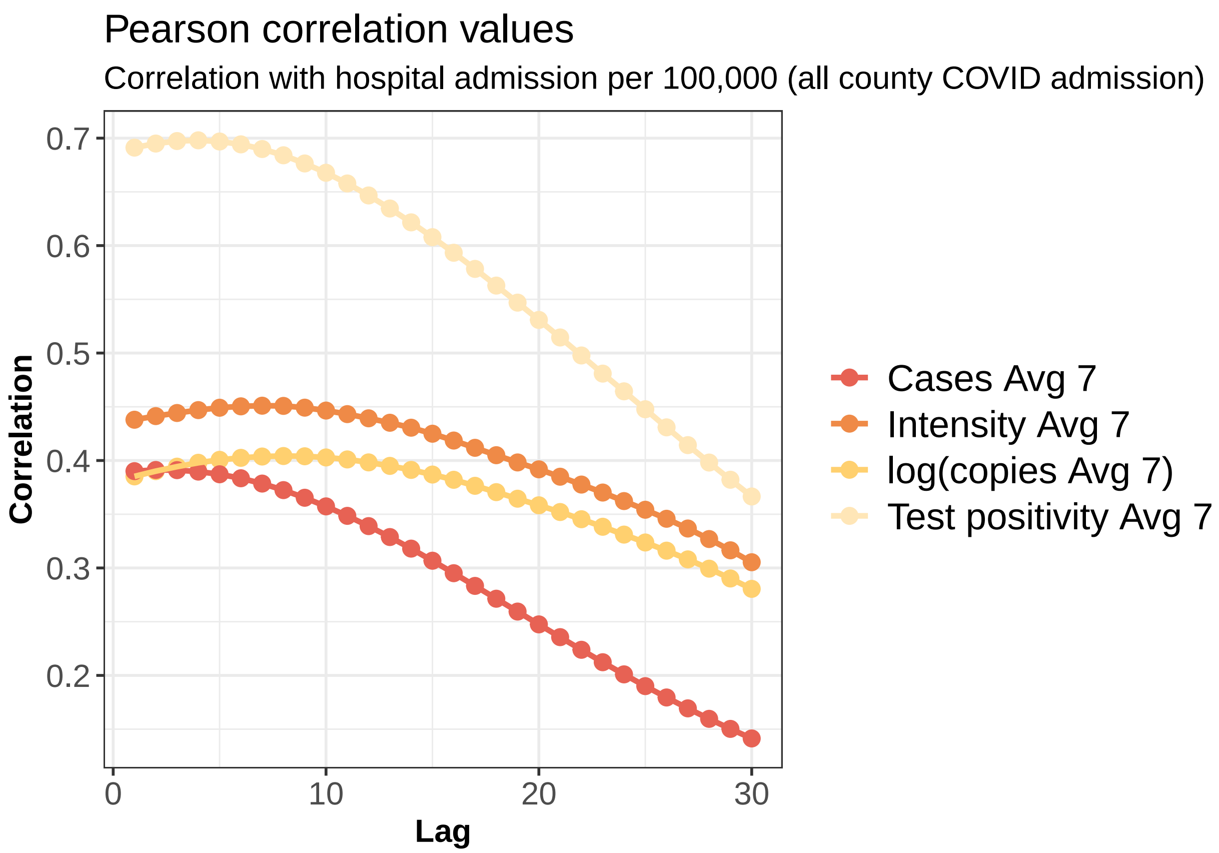

0.3.2 Correlation results across labs

Individual correlations within each lab method

```
# all hospital admisstions
data.county <- readRDS("E:/Dropbox/CEMI/Wastewater/Papers/Modelling hospitalizations/Data/county_data_ho
data.county$intensity.7avg <- data.county$intensity.approx.7avg
data.county$sars2.7avg <- data.county$sars2.approx.7avg

# create two sets and see which one linearly correlates the best? could return to this maybe
data.county <- data.county %>%
  arrange(-desc(date))%>%
  group_by(county) %>%
  mutate(
    intensity.7avg_lag_10 = lag(intensity.7avg, 10, default = NA),
    sars2.7avg_lag_10 = lag(sars2.7avg, 10, default = NA)

  ) %>%
  ungroup() %>%
  filter(!county == "Allegany")

# per 100k hos admissions
data.county$hos.7avg_100 <- data.county$county.prop * data.county$hos.7avg

pal <- met.brewer(name= "Hiroshige", n = 5)
```

```
cor_plot_function <- function(lab_name, ylim){

  # calculate correlation
  data_lab <- data.county %>% filter(lab_id_r == lab_name)
  cor_rawcopies_lab <- cor(data_lab$hos.7avg_100, log(data_lab$sars2.7avg_lag_10), use = "na.or.complete

  # plot
  cor_plot <-
    ggplot(data = data.county %>% filter(lab_id_r == lab_name))+
    geom_point(aes(x = log(sars2.7avg_lag_10), y = (hos.7avg_100)), alpha = 0.6, color = "#ef8a47")+
    #facet_wrap(~lab_id_r, scale = "free")+
    geom_smooth(aes(x = log(sars2.7avg_lag_10), y = (hos.7avg_100)), method = "lm", se=FALSE, color = "
                size = 1.5)+
    labs(title = lab_name,
         subtitle = paste("Pearson correlation ", round(cor_rawcopies_lab, 3)),
         x  = "",
         y = "")+
    theme_minimal()+
    theme(axis.text=element_text(size=10),
          axis.title=element_text(size=10,face="bold"),
          plot.title = element_text(size=11),
          plot.subtitle = element_text(size = 9),
          plot.caption = element_text(size = 8),
          legend.text = element_text(size = 10),
          legend.title = element_blank())+
    lims(y = c(0,ylim))
  # return the plot
  return(cor_plot)
}
quadrant_cor <- cor_plot_function(lab_name = "Quadrant", ylim = 8)

stonybrook_cor <- cor_plot_function(lab_name = "Stony Brook", ylim = 6)

nyc_cor <- cor_plot_function(lab_name = "NYC", ylim = 5)

ub_cor <- cor_plot_function(lab_name = "UB-SUNY 1", ylim = 5)

ub_cor2 <- cor_plot_function(lab_name = "UB-SUNY 2", ylim = 8)

# all labs
# calculate correlation
data_lab <- data.county %>% filter(!lab_id_r == "GO-Health")
cor_rawcopies_lab <- cor(data_lab$hos.7avg_100, log(data_lab$sars2.7avg_lag_10), use = "na.or.complete")

# plot
cor_plot <-
  ggplot(data = data_lab)+
  geom_point(aes(x = log(sars2.7avg_lag_10), y = (hos.7avg_100)), alpha = 0.6, color = "#ef8a47")+
  #facet_wrap(~lab_id_r, scale = "free")+
  geom_smooth(aes(x = log(sars2.7avg_lag_10), y = (hos.7avg_100)), method = "lm", se=FALSE, color = "#3
              size = 1.5)+
  labs(title = "All labs",
       subtitle = paste("Pearson correlation ", round(cor_rawcopies_lab, 3)),
```

```
       x  = "",
       y = "")+
  theme_minimal()+
    theme(axis.text=element_text(size=10),
          axis.title=element_text(size=10,face="bold"),
          plot.title = element_text(size=11),
          plot.subtitle = element_text(size = 9),
          plot.caption = element_text(size = 8),
          legend.text = element_text(size = 10),
          legend.title = element_blank())+
  ylim(0,18)

# all labs
# calculate correlation
data_lab <- data.county %>% filter(lab_id_r == "UB-SUNY 2")
data_lab <- data_lab %>% filter(!county == "Allegany")
cor_rawcopies_ub <- cor(data_lab$hos.7avg_100, log(data_lab$sars2.approx.7avg), use = "na.or.complete")

# plot
ub_plot2 <-
  ggplot(data = data_lab)+
  geom_point(aes(x = log(sars2.7avg_lag_10), y = (hos.7avg_100)), alpha = 0.6, color = "#ef8a47")+
  #facet_wrap(~lab_id_r, scale = "free")+
  geom_smooth(aes(x = log(sars2.7avg_lag_10), y = (hos.7avg_100)), method = "lm", se=FALSE, color = "#3
             size = 1.5)+
  labs(title = "UB-SUNY 2",
       subtitle = paste("Pearson correlation ", round(cor_rawcopies_ub, 3)),
       x  = "",
       y = "")+
  theme_minimal()+
    theme(axis.text=element_text(size=10),
          axis.title=element_text(size=10,face="bold"),
          plot.title = element_text(size=11),
          plot.subtitle = element_text(size = 9),
          plot.caption = element_text(size = 8),
          legend.text = element_text(size = 10),
          legend.title = element_blank())+
  ylim(0,8)


# panel
#combine using cowplot
panel <-cowplot::plot_grid(nyc_cor, quadrant_cor, stonybrook_cor, ub_cor, ub_plot2, cor_plot, nrow = 3,
                           align='vh', vjust=1, scale = 1)

#create common x and y labels
y.grob <- textGrob("Hospital admissions per 100k",
                   gp=gpar(fontface="bold", col="black", fontsize=12), rot=90)

x.grob <- textGrob("ln(raw gene copies)",
                   gp=gpar(fontface="bold", col="black", fontsize=12))

title <- textGrob("Correlations for ln(raw gene copies) and hospital admissions per 100k",
```

```
                       gp = gpar(fontface = "bold", col = "black", fontsize = 13))

# add to plot
grid.arrange(arrangeGrob(panel, left = y.grob, bottom = x.grob, top = title))
```

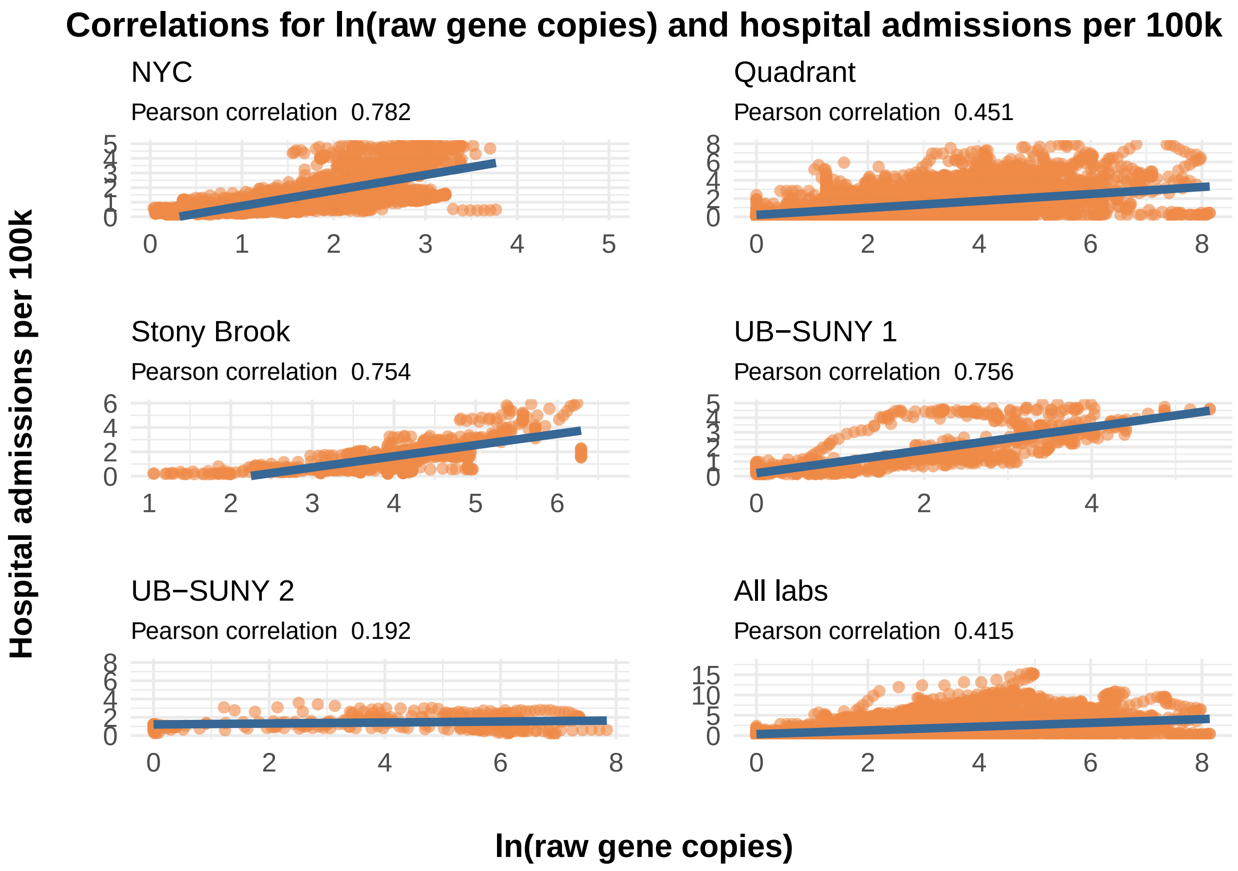

0.4 Model fitting

We fit a generalized linear mixed model (GLMM) using the glmmTMB R package. We started out with a Poisson distribution, then tried other adjustments including negative binomial and zero-inflated models.

The Poisson model with random intercepts for labs and counties proved to be the most accurate based on the lowest mean absolute scaled error (MASE) values. Below, I present several models, their output, and MASE values.

0.4.1 Training and testing datasets for each lab

0.5 Final model selected

```
# covariates
all_labs_cov <- glmmTMB(hos.7avg_100 ~ #(log(sars2.7avg_lag_10))  +
                                scale(hos.count_sample_day_10) +lab_id_r+
                                scale(CDKRateA) +
```

```
                                   scale(BMI30Rate) +
                                   scale(svi_county) +
                                   scale(over_50_per)+
                                   # holidays a is major, b is minor, c is no holiday
                                   category +
                                   (1|county) ,
                                 data = new_training,
                                 family = poisson,
                                 control=glmmTMBControl(optimizer=optim, optArgs=list(method="BFGS"))
)
summary(all_labs_cov)
```

0.5.0.1 Model with covariates only

```
##  Family: poisson  ( log )
## Formula:
## hos.7avg_100 ~ scale(hos.count_sample_day_10) + lab_id_r + scale(CDKRateA) +
##     scale(BMI30Rate) + scale(svi_county) + scale(over_50_per) +
##     category + (1 | county)
## Data: new_training
##
##      AIC      BIC   logLik deviance df.resid
##   9015.6   9097.0  -4494.8   8989.6     3866
##
## Random effects:
##
## Conditional model:
##  Groups Name        Variance Std.Dev.
##  county (Intercept) 0.222    0.4712
## Number of obs: 3879, groups:  county, 47
##
## Conditional model:
##                                 Estimate Std. Error z value Pr(>|z|)
## (Intercept)                     -0.35657    0.31378  -1.136  0.25579
## scale(hos.count_sample_day_10)  0.36403    0.02347  15.511  < 2e-16 ***
## lab_id_rQuadrant                 0.71572    0.34062   2.101  0.03562 *
## lab_id_rStony Brook             -0.35334    0.57971  -0.610  0.54219
## lab_id_rUB-SUNY 1                0.12614    0.41973   0.301  0.76378
## lab_id_rUB-SUNY 2                0.79902    0.40944   1.952  0.05100 .
## scale(CDKRateA)                  0.18403    0.09361   1.966  0.04930 *
## scale(BMI30Rate)                 0.07721    0.08639   0.894  0.37144
## scale(svi_county)               -0.06991    0.08827  -0.792  0.42837
## scale(over_50_per)               0.23558    0.08701   2.708  0.00678 **
## categoryb                       -0.27622    0.04931  -5.602 2.12e-08 ***
## categoryc                       -0.08050    0.03694  -2.180  0.02929 *
## ---
## Signif. codes:  0 '***' 0.001 '**' 0.01 '*' 0.05 '.' 0.1 ' ' 1
```

```
cov_summary <- coef_geography_function(model = all_labs_cov, testing_data = new_model_testing1)
```

```r
# vaccines
all_labs_vax <- glmmTMB(hos.7avg_100 ~
                          scale(hos.count_sample_day_10) +lab_id_r+
                          scale(Fully_vaccinated_county_prop) +
                          scale(CDKRateA) +
                          scale(BMI30Rate) +
                          # svi
                          scale(svi_county) +
                          scale(over_50_per)+
                          # holidays a is major, b is minor, c is no holiday
                          category +
                          (1|county) ,
                        data = new_training,
                        family = poisson,
                        control=glmmTMBControl(optimizer=optim, optArgs=list(method="BFGS"))
)
summary(all_labs_vax)
```

0.5.0.2 Model with vaccination data

```
##  Family: poisson  ( log )
## Formula:
## hos.7avg_100 ~ scale(hos.count_sample_day_10) + lab_id_r + scale(Fully_vaccinated_county_prop) +
##     scale(CDKRateA) + scale(BMI30Rate) + scale(svi_county) +
##     scale(over_50_per) + category + (1 | county)
## Data: new_training
##
##      AIC      BIC   logLik deviance df.resid
##   9016.5   9104.2  -4494.2   8988.5     3865
##
## Random effects:
##
## Conditional model:
##  Groups Name        Variance Std.Dev.
##  county (Intercept) 0.2      0.4472
## Number of obs: 3879, groups:  county, 47
##
## Conditional model:
##                                     Estimate Std. Error z value Pr(>|z|)
## (Intercept)                         -0.28508    0.30570  -0.933   0.3511
## scale(hos.count_sample_day_10)       0.36568    0.02348  15.577  < 2e-16 ***
## lab_id_rQuadrant                     0.63920    0.33159   1.928   0.0539 .
## lab_id_rStony Brook                 -0.43305    0.55659  -0.778   0.4365
## lab_id_rUB-SUNY 1                    0.04183    0.40762   0.103   0.9183
## lab_id_rUB-SUNY 2                    0.71597    0.39684   1.804   0.0712 .
## scale(Fully_vaccinated_county_prop) -0.10158    0.09209  -1.103   0.2700
## scale(CDKRateA)                      0.19068    0.08939   2.133   0.0329 *
## scale(BMI30Rate)                     0.04259    0.08819   0.483   0.6291
## scale(svi_county)                   -0.10291    0.08930  -1.152   0.2492
## scale(over_50_per)                   0.23300    0.08282   2.813   0.0049 **
## categoryb                           -0.27668    0.04931  -5.611 2.01e-08 ***
## categoryc                           -0.08065    0.03693  -2.184   0.0290 *
```

```
## ---
## Signif. codes:  0 '***' 0.001 '**' 0.01 '*' 0.05 '.' 0.1 ' ' 1
```

```
vax_summary <- coef_geography_function(model = all_labs_vax, testing_data = new_model_testing1)
```

```
# test positivity
all_labs_tp <- glmmTMB(hos.7avg_100 ~
                         scale(hos.count_sample_day_10) +lab_id_r+
                         scale(log1p(test_pos.7avg_lag_10)) +
                         # vaccines
                         scale(Fully_vaccinated_county_prop) +
                         scale(CDKRateA) +
                         scale(BMI30Rate) +
                         # svi
                         scale(svi_county) +
                         # demographics
                         scale(over_50_per)+
                         # holidays a is major, b is minor, c is no holiday
                         category +
                         #season_4 +
                         (1|county) ,
                       data = new_training,
                       family = poisson,
                       control=glmmTMBControl(optimizer=optim, optArgs=list(method="BFGS"))
)
summary(all_labs_tp)
```

0.5.0.3 Model with test positivity

```
##  Family: poisson  ( log )
## Formula:
## hos.7avg_100 ~ scale(hos.count_sample_day_10) + lab_id_r + scale(log1p(test_pos.7avg_lag_10)) +
##     scale(Fully_vaccinated_county_prop) + scale(CDKRateA) + scale(BMI30Rate) +
##     scale(svi_county) + scale(over_50_per) + category + (1 |      county)
## Data: new_training
##
##      AIC      BIC   logLik deviance df.resid
##   8711.8   8805.7  -4340.9   8681.8     3864
##
## Random effects:
##
## Conditional model:
##  Groups Name        Variance Std.Dev.
##  county (Intercept) 0.1881   0.4337
## Number of obs: 3879, groups:  county, 47
##
## Conditional model:
##                                      Estimate Std. Error z value Pr(>|z|)
## (Intercept)                          0.051915   0.296848   0.175   0.8612
## scale(hos.count_sample_day_10)       0.178776   0.026507   6.744 1.54e-11 ***
```

```
## lab_id_rQuadrant                      0.041743   0.323323   0.129   0.8973
## lab_id_rStony Brook                  -0.422289   0.540858  -0.781   0.4349
## lab_id_rUB-SUNY 1                    -0.199236   0.396023  -0.503   0.6149
## lab_id_rUB-SUNY 2                    -0.293151   0.389090  -0.753   0.4512
## scale(log1p(test_pos.7avg_lag_10))    0.382888   0.022121  17.309  < 2e-16 ***
## scale(Fully_vaccinated_county_prop) -0.231400   0.087115  -2.656   0.0079 **
## scale(CDKRateA)                       0.186174   0.086951   2.141   0.0323 *
## scale(BMI30Rate)                     -0.055225   0.085855  -0.643   0.5201
## scale(svi_county)                    -0.096051   0.086615  -1.109   0.2675
## scale(over_50_per)                    0.151109   0.080548   1.876   0.0607 .
## categoryb                             0.009857   0.051829   0.190   0.8492
## categoryc                             0.051471   0.037395   1.376   0.1687
## ---
## Signif. codes:  0 '***' 0.001 '**' 0.01 '*' 0.05 '.' 0.1 ' ' 1
```

```
tp_summary <- coef_geography_function(model = all_labs_tp, testing_data = new_model_testing1)
```

```
# full model
all_labs_ww <- glmmTMB(hos.7avg_100 ~ scale(log(sars2.7avg_lag_10))  +
                         scale(hos.count_sample_day_10) +lab_id_r+
                         scale(log1p(test_pos.7avg_lag_10)) +
                         scale(Fully_vaccinated_county_prop) +
                         scale(CDKRateA) +
                         scale(BMI30Rate) +
                         scale(svi_county) +
                         scale(over_50_per)+
                         # holidays a is major, b is minor, c is no holiday
                         category +
                         #season_4 +
                         (1|county) ,
                       data = new_training,
                       family = poisson,
                       control=glmmTMBControl(optimizer=optim, optArgs=list(method="BFGS"))
)
summary(all_labs_ww)
```

0.5.0.4 FINAL MODEL WITH ALL VARIABLES

```
##  Family: poisson  ( log )
## Formula:
## hos.7avg_100 ~ scale(log(sars2.7avg_lag_10)) + scale(hos.count_sample_day_10) +
##     lab_id_r + scale(log1p(test_pos.7avg_lag_10)) + scale(Fully_vaccinated_county_prop) +
##     scale(CDKRateA) + scale(BMI30Rate) + scale(svi_county) +
##     scale(over_50_per) + category + (1 | county)
## Data: new_training
##
##      AIC      BIC   logLik deviance df.resid
##   8697.4   8797.6  -4332.7   8665.4     3863
##
## Random effects:
```

```
##
## Conditional model:
##  Groups Name        Variance Std.Dev.
##  county (Intercept) 0.1926   0.4389
## Number of obs: 3879, groups:  county, 47
##
## Conditional model:
##                                       Estimate Std. Error z value Pr(>|z|)
## (Intercept)                           0.144404   0.300977   0.480  0.63138
## scale(log(sars2.7avg_lag_10))         0.088554   0.021870   4.049 5.14e-05 ***
## scale(hos.count_sample_day_10)        0.170910   0.026698   6.402 1.54e-10 ***
## lab_id_rQuadrant                     -0.056233   0.327865  -0.172  0.86382
## lab_id_rStony Brook                  -0.545793   0.547797  -0.996  0.31908
## lab_id_rUB-SUNY 1                    -0.168546   0.400292  -0.421  0.67371
## lab_id_rUB-SUNY 2                    -0.424009   0.394833  -1.074  0.28287
## scale(log1p(test_pos.7avg_lag_10))    0.352909   0.023354  15.111  < 2e-16 ***
## scale(Fully_vaccinated_county_prop) -0.236765   0.088160  -2.686  0.00724 **
## scale(CDKRateA)                       0.195282   0.087956   2.220  0.02640 *
## scale(BMI30Rate)                     -0.044702   0.086874  -0.515  0.60686
## scale(svi_county)                    -0.106215   0.087644  -1.212  0.22555
## scale(over_50_per)                    0.157029   0.081468   1.927  0.05392 .
## categoryb                             0.005873   0.051763   0.113  0.90966
## categoryc                             0.041605   0.037443   1.111  0.26651
## ---
## Signif. codes:  0 '***' 0.001 '**' 0.01 '*' 0.05 '.' 0.1 ' ' 1
```

```
ww_summary <- coef_geography_function(model = all_labs_ww, testing_data = new_model_testing1)
```

```
# ww only
all_labs_ww_only <- glmmTMB(hos.7avg_100 ~ scale(log(sars2.7avg_lag_10))  +
                         scale(hos.count_sample_day_10) +lab_id_r+
                         scale(Fully_vaccinated_county_prop) +
                         scale(CDKRateA) +
                         scale(BMI30Rate) +
                         # svi
                         scale(svi_county) +
                         # demographics
                         scale(over_50_per)+
                         # holidays a is major, b is minor, c is no holiday
                         category +
                         #season_4 +
                         (1|county) ,
                       data = new_training,
                       family = poisson,
                       control=glmmTMBControl(optimizer=optim, optArgs=list(method="BFGS"))
)
summary(all_labs_ww_only)
```

0.5.0.5 MODEL WITH WASTEWATER DATA BUT NOT TEST POSITIVITY DATA

```
##  Family: poisson  ( log )
```

```
## Formula:
## hos.7avg_100 ~ scale(log(sars2.7avg_lag_10)) + scale(hos.count_sample_day_10) +
##     lab_id_r + scale(Fully_vaccinated_county_prop) + scale(CDKRateA) +
##     scale(BMI30Rate) + scale(svi_county) + scale(over_50_per) +
##     category + (1 | county)
## Data: new_training
##
##      AIC      BIC   logLik deviance df.resid
##   8926.4   9020.3  -4448.2   8896.4     3864
##
## Random effects:
##
## Conditional model:
##  Groups Name        Variance Std.Dev.
##  county (Intercept) 0.216    0.4648
## Number of obs: 3879, groups:  county, 47
##
## Conditional model:
##                                     Estimate Std. Error z value Pr(>|z|)
## (Intercept)                         -0.02078    0.31762  -0.065   0.9478
## scale(log(sars2.7avg_lag_10))        0.19466    0.02038   9.550  < 2e-16 ***
## scale(hos.count_sample_day_10)       0.31586    0.02444  12.923  < 2e-16 ***
## lab_id_rQuadrant                     0.31724    0.34521   0.919   0.3581
## lab_id_rStony Brook                 -0.70784    0.57793  -1.225   0.2207
## lab_id_rUB-SUNY 1                    0.06083    0.42163   0.144   0.8853
## lab_id_rUB-SUNY 2                    0.25157    0.41423   0.607   0.5436
## scale(Fully_vaccinated_county_prop) -0.13214    0.09383  -1.408   0.1590
## scale(CDKRateA)                      0.20837    0.09273   2.247   0.0246 *
## scale(BMI30Rate)                     0.04879    0.09133   0.534   0.5932
## scale(svi_county)                   -0.12639    0.09252  -1.366   0.1719
## scale(over_50_per)                   0.23086    0.08590   2.688   0.0072 **
## categoryb                           -0.23408    0.04932  -4.746 2.07e-06 ***
## categoryc                           -0.07945    0.03679  -2.159   0.0308 *
## ---
## Signif. codes:  0 '***' 0.001 '**' 0.01 '*' 0.05 '.' 0.1 ' ' 1
```

```
performance::r2(all_labs_ww_only)
```

```
## # R2 for Mixed Models
##
##   Conditional R2: 0.421
##      Marginal R2: 0.213
```

```
ww_only_summary <- coef_geography_function(model = all_labs_ww_only, testing_data = new_model_testing1)
```

0.6 Correlation between predicted and observed

```
# NEW MODEL PREDICTIONS - MARCH to JUNE
# NEW MODEL
```

```r
# NEW MODEL
# create new testing and training datasets from the main data.county file
data.county <- readRDS("E:/Dropbox/CEMI/Wastewater/Papers/Modelling hospitalizations/Data/county_data_ho
data.county$sars2.7avg <- data.county$sars2.approx.7avg
data.county$intensity.7avg <- data.county$intensity.approx.7avg

# lags
data.county <- data.county %>%
  arrange(-desc(date))%>%
  group_by(county) %>%
  mutate(
    intensity.7avg_lag_10 = lag(intensity.7avg, 10, default = NA),
    sars2.7avg_lag_10 = lag(sars2.7avg, 10, default = NA),
    test_pos.7avg_lag_10 = lag(test.pos.avg7, default = NA),
    hos.count_sample_day_10 = lag(hos.count, default = NA)

  ) %>%
  ungroup()
data.county$hos.7avg_100 <- data.county$hos.7avg * data.county$county.prop

# testing and training datasets - all labs

# out of data: queens, broome, oswego, albany, Chautauqua
# also hold out June 2022*
counties_out <- c("Queens", "Broome", "Oswego", "Albany", "Chautauqua")

new_model_training <- data.county %>%
  filter(!county %in% counties_out) %>%
  filter(date<= "2022-06-01" & date >= "2022-03-01")
new_model_testing1 <- data.county %>%
  #filter(county %in% counties_out)%>%
  filter(!lab_id_r == "GO-Health") %>%
  filter(date<= "2022-06-30" & date >= "2022-03-01")

new_model_testing2 <- data.county %>%
  filter(county %in% counties_out)%>%
  filter(date<= "2022-06-30" & date >= "2022-03-01")

# remove go health
new_training <- new_model_training %>% filter(!lab_id_r == "GO-Health") %>%
  filter(!is.na(sars2.7avg_lag_10))

# full model
all_labs_ww <- glmmTMB(hos.7avg_100 ~ (log(sars2.7avg_lag_10))  +
                         (hos.count_sample_day_10) +lab_id_r+
                         (log1p(test_pos.7avg_lag_10)) +
                         # vaccines
                         #Partially_vaccinated_county_prop +
                         (Fully_vaccinated_county_prop) +
                         # comorbidities
                         #CancerRate +
                         #AsthmaRate +
                         (CDKRateA) +
```

```r
                          #RespRate +
                          (BMI30Rate) +
                          # svi
                          (svi_county) +
                          # demographics
                          (over_50_per)+
                          # holidays a is major, b is minor, c is no holiday
                          category +
                          #season_4 +
                          (1|county) ,
                        data = new_training,
                        family = poisson,
                        control=glmmTMBControl(optimizer=optim, optArgs=list(method="BFGS"))
)

# cor plots for predict v. observed
# original model predictions
new_all_labs_data <- new_model_testing1 %>%
  filter(!is.na(sars2.7avg_lag_10) & !is.na(hos.count_sample_day_10) & !is.na(test_pos.7avg_lag_10))
predict_copies_8 <- predict(all_labs_ww, newdata = new_all_labs_data, type = "response", se.fit = TRUE,
                            prediction = "interval")

# make a dataframe
df_predictions <- as.data.frame(predict_copies_8)

df_predictions$LL <- df_predictions$fit - 1.96 * df_predictions$se.fit
df_predictions$UL <- df_predictions$fit + 1.96 * df_predictions$se.fit

head(df_predictions)
```

0.6.0.1 In data predictions (testing dataset)

```
##          fit     se.fit         LL        UL
## 1 0.82852255 0.09525062 0.64183134 1.0152138
## 2 1.37647156 0.11032656 1.16023151 1.5927116
## 3 1.05836950 0.09281375 0.87645454 1.2402845
## 4 0.09934633 0.02950948 0.04150775 0.1571849
## 5 1.41635480 0.12102948 1.17913701 1.6535726
## 6 0.90739371 0.08537972 0.74004946 1.0747380
```

```r
# bind to data
new_all_labs_data <- cbind(new_all_labs_data, df_predictions)
new_all_labs_data$mean_dif <- new_all_labs_data$hos.7avg_100 - new_all_labs_data$fit

# In data predictions
cor_in_8 <-  grobTree(textGrob(paste("Pearson Correlation : ", round(cor(new_all_labs_data$hos.7avg_100
                                                                       new_all_labs_data$fit, method =
                                x = 0.07, y = 0.9, hjust = 0,
                                gp = gpar(col = "black", fontsize = 11, fontface = "bold")))

pred.v.obs_plot <-
  ggplot(data = new_all_labs_data, aes( x = hos.7avg_100, y = fit))+
  geom_point(color = "#e76254", alpha = 0.6)+
```

```
  theme(legend.position = "bottom",
        panel.background = element_rect(fill = "white",
                                        colour = "white",
                                        size = 0.5, linetype = "solid"),
        panel.grid.major.y = element_line(size = 0.5, linetype = 'solid',
                                          colour = "grey"),
        axis.text=element_text(size=12),
        axis.title=element_text(size=12,face="bold"),
        plot.title = element_text(size=15),
        plot.subtitle = element_text(size = 12),
        legend.text = element_text(size = 12)
  )+
  labs(title = "A. Observed v. predicted values (in-data predictions)",
       subtitle = "New hospital admissions per 100k population\nCounty model for all labs and raw gene c
       x = "Observed",
       y = "Predicted")+
  annotation_custom(cor_in_8)+
  lims(x = c(0, 5),
       y = c(0,5))+
  geom_smooth(method = "lm", se = FALSE, color = "#376795",
              size = 1.5)

pred.v.obs_plot
```

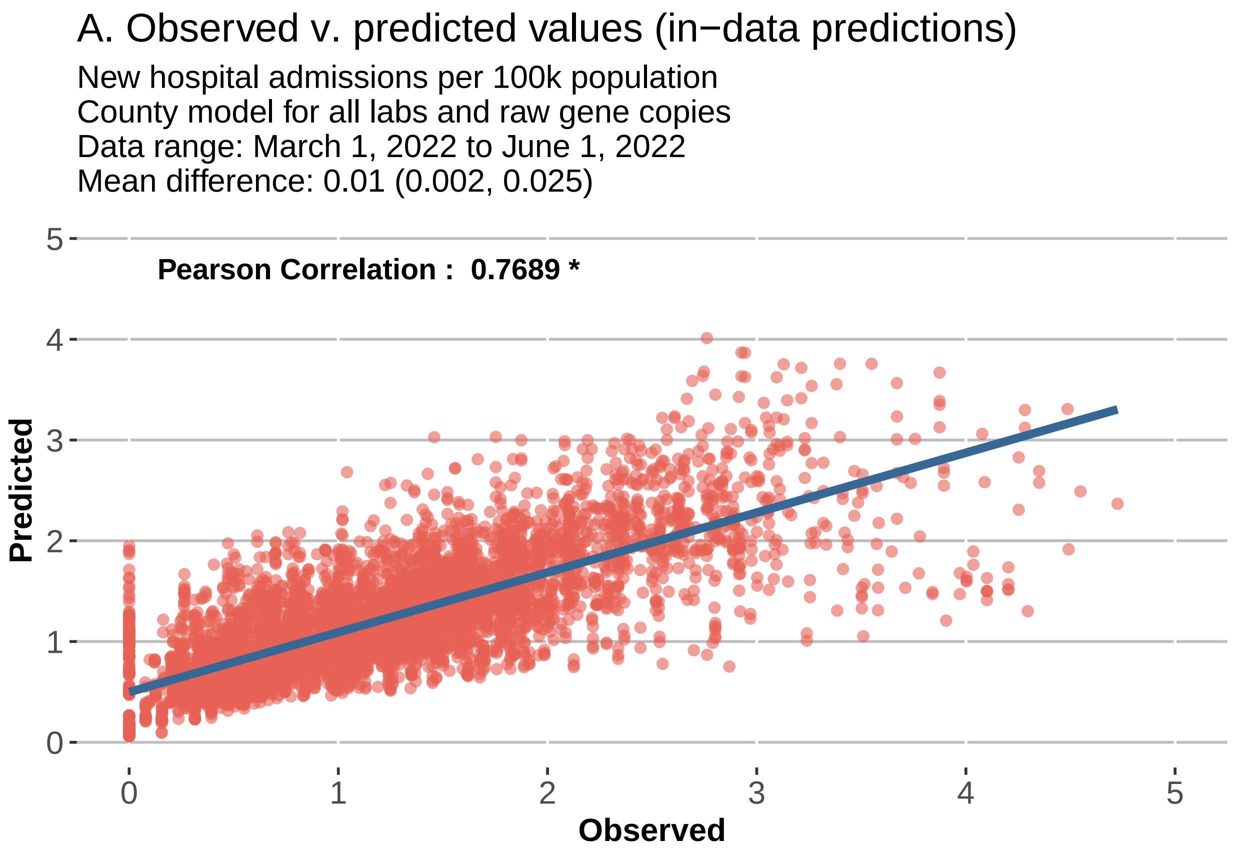

```r
# new dataset
new_all_labs_data2 <- new_model_testing2 %>%
    filter(!is.na(sars2.7avg_lag_10) & !is.na(hos.count_sample_day_10) & !is.na(test_pos.7avg_lag_10))
predict_copies_8 <- predict(all_labs_ww, newdata = new_all_labs_data2, type = "response",
    se.fit = TRUE, prediction = "interval")

# make a dataframe
df_predictions <- as.data.frame(predict_copies_8)

df_predictions$LL <- df_predictions$fit - 1.96 * df_predictions$se.fit
df_predictions$UL <- df_predictions$fit + 1.96 * df_predictions$se.fit

# bind to data
new_all_labs_data2 <- cbind(new_all_labs_data2, df_predictions)
new_all_labs_data2$mean_dif <- new_all_labs_data2$hos.7avg_100 - new_all_labs_data2$fit

mean <- mean(new_all_labs_data2$mean_dif)
se <- sd(new_all_labs_data2$mean_dif)/length(new_all_labs_data2$date)
LL <- mean - 1.96 * se
UL <- mean + 1.96 * se

# correlation
cor_in_8 <- grobTree(textGrob(paste("Pearson Correlation : ", round(cor(new_all_labs_data2$hos.7avg_100
    new_all_labs_data2$fit, method = "pearson", use = "complete.obs"), 4), "*"),
    x = 0.07, y = 0.9, hjust = 0, gp = gpar(col = "black", fontsize = 11, fontface = "bold")))

# plot 2
pred.v.obs_plot2 <- ggplot(data = new_all_labs_data2, aes(x = hos.7avg_100, y = fit)) +
    geom_point(color = "#e76254", alpha = 0.6) + theme(legend.position = "bottom",
    panel.background = element_rect(fill = "white", colour = "white", size = 0.5,
        linetype = "solid"), panel.grid.major.y = element_line(size = 0.5, linetype = "solid",
        colour = "grey"), axis.text = element_text(size = 12), axis.title = element_text(size = 12,
        face = "bold"), plot.title = element_text(size = 15), plot.subtitle = element_text(size = 12),
    legend.text = element_text(size = 12)) + labs(title = "B. Observed v. predicted values (out-of-data
    subtitle = "New hospital admissions per 100k population\nCounty model for all labs and raw gene cop
    x = "Observed", y = "Predicted") + annotation_custom(cor_in_8) + lims(x = c(0,
    5), y = c(0, 5)) + geom_smooth(method = "lm", se = FALSE, color = "#376795",
    size = 1.5)

pred.v.obs_plot2
```

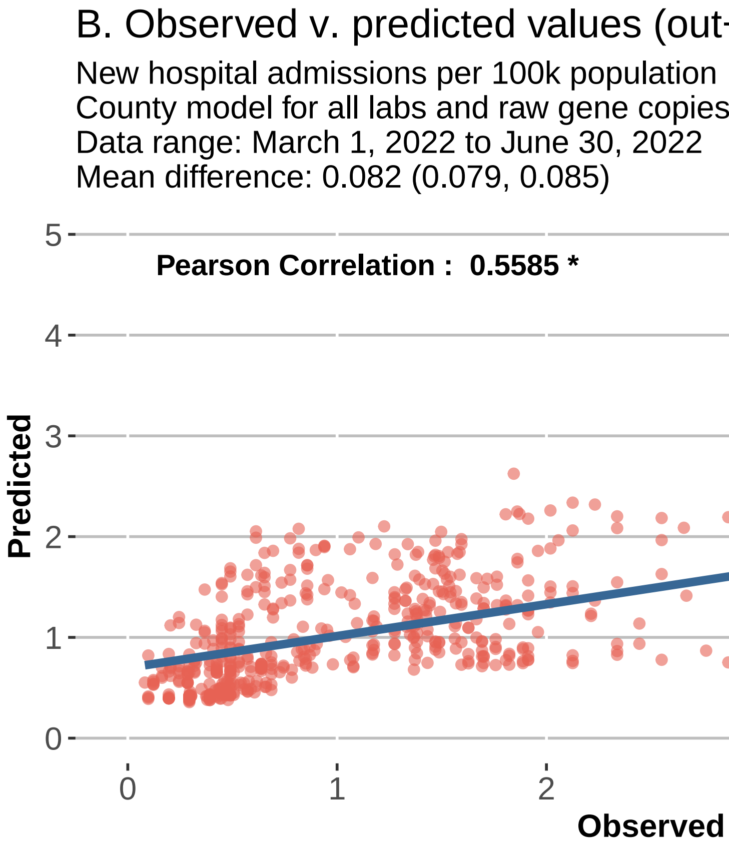

0.6.0.2 Out of data predictions - validation dataset

0.7 County example plots

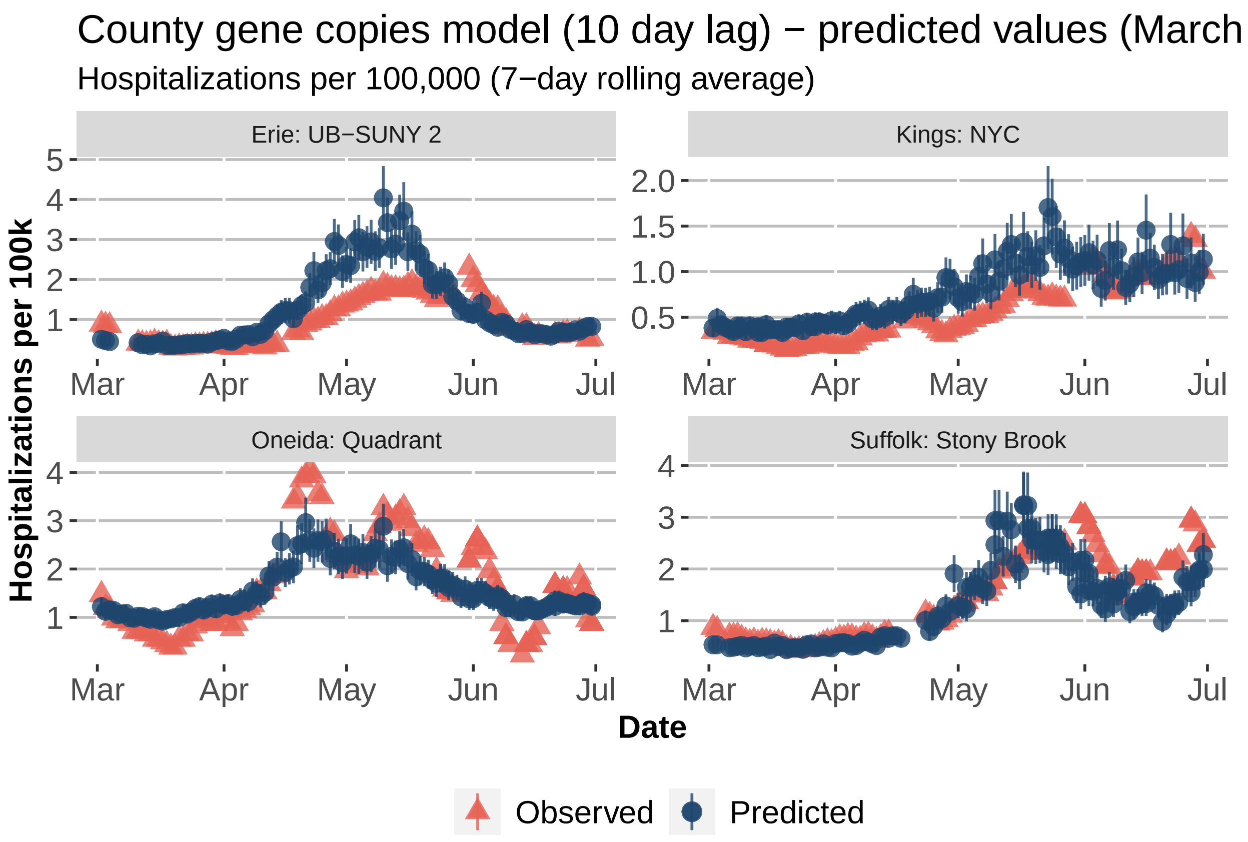

Supplement: Multimedia component 2 [file mmc2.pdf]
